# Muscle-driven forward dynamic active hybrid model of the lumbosacral spine: combined FEM and multibody simulation

**DOI:** 10.3389/fbioe.2023.1223007

**Published:** 2023-09-27

**Authors:** Robin Remus, Sascha Selkmann, Andreas Lipphaus, Marc Neumann, Beate Bender

**Affiliations:** ^1^ Chair of Product Development, Department of Mechanical Engineering, Ruhr-University Bochum, Bochum, Germany; ^2^ Biomechanics Research Group, Chair of Product Development, Department of Mechanical Engineering, Ruhr-University Bochum, Bochum, Germany

**Keywords:** finite element model, musculoskeletal multibody model, muscle-driven, forward dynamics, spinal stability, active hybrid model, motion capture, lumbar spine

## Abstract

Most spine models belong to either the musculoskeletal multibody (MB) or finite element (FE) method. Recently, coupling of MB and FE models has increasingly been used to combine advantages of both methods. Active hybrid FE-MB models, still rarely used in spine research, avoid the interface and convergence problems associated with model coupling. They provide the inherent ability to account for the full interplay of passive and active mechanisms for spinal stability. In this paper, we developed and validated a novel muscle-driven forward dynamic active hybrid FE-MB model of the lumbosacral spine (LSS) in ArtiSynth to simultaneously calculate muscle activation patterns, vertebral movements, and internal mechanical loads. The model consisted of the rigid vertebrae L1-S1 interconnected with hyperelastic fiber-reinforced FE intervertebral discs, ligaments, facet joints, and force actuators representing the muscles. Morphological muscle data were implemented via a semi-automated registration procedure. Four auxiliary bodies were utilized to describe non-linear muscle paths by wrapping and attaching the anterior abdominal muscles. This included an abdominal plate whose kinematics was optimized using motion capture data from upper body movements. Intra-abdominal pressure was calculated from the forces of the abdominal muscles compressing the abdominal cavity. For the muscle-driven approach, forward dynamics assisted data tracking was used to predict muscle activation patterns that generate spinal postures and balance the spine without prescribing accurate spinal kinematics. During calibration, the maximum specific muscle tension and spinal rhythms resulting from the model dynamics were evaluated. To validate the model, load cases were simulated from −10° extension to +30° flexion with weights up to 20 kg in both hands. The biomechanical model responses were compared with *in vivo* literature data of intradiscal pressures, intra-abdominal pressures, and muscle activities. The results demonstrated high agreement with this data and highlight the advantages of active hybrid modeling for the LSS. Overall, this new self-contained tool provides a robust and efficient estimation of LSS biomechanical responses under *in vivo* similar loads, for example, to improve pain treatment by spinal stabilization therapies.

## 1 Introduction

Low back pain is a leading cause of severe activity limitations in the personal and professional lifes of sufferers (Duthey, 2013) and affects most adults during their lifetime (Andersson, 1998; Setchell et al., 2019). Many of the underlying causes and mechanisms that lead to back pain are not yet fully understood (Panjabi, 2006; Reeves et al., 2019). With the complexity of the interplay between a variety of anatomical structures, the lumbar spine is an important topic of biomechanical research (Martin et al., 2018). To increase our understanding, a wide range of high-quality *in vivo* and *in vitro* spine studies have been performed over the past decades (Sato et al., 1999; Wilke et al., 2001; Heuer et al., 2007; Oxland, 2016). Even latest experimental methods, however, are reaching their limits in studying internal mechanics like muscle forces or stress states in soft tissues, the overall context of the stabilizing functions of individual muscles (Santaguida and McGill, 1995; Freeman et al., 2010; Arbanas et al., 2013), and thus the possible causes of pain (Panjabi, 2003; Dreischarf et al., 2015; Hodges et al., 2015; Reeves et al., 2019). Also, in terms of cost reduction, ethical justifiability, and broader patient populations, numerical simulation methods are increasingly being used with varying focus, level of detail, and solution approach (Galbusera and Wilke, 2018; Knapik et al., 2022).

Most validated and extensively used biomechanical spine models belong to either the musculoskeletal multibody (MB) (de Zee et al., 2007; Christophy et al., 2012; Bruno et al., 2015; Senteler et al., 2016; Malakoutian et al., 2018; Bayoglu et al., 2019; Lerchl et al., 2022; Meszaros-Beller et al., 2023) or implicit finite element (FE) method (Schmidt et al., 2006; Ayturk and Puttlitz, 2011; Dreischarf et al., 2014; Naserkhaki et al., 2018; Turbucz et al., 2022). Musculoskeletal MB models are used to analyze mechanisms consisting of rigid bodies subject to mechanically simplifying joints and constraints. Studies thus examined interindividual muscle activation patterns, joint reaction forces, or vertebral movements in varying postures during different activities. Resulting muscle redundancy problems are commonly solved inverse dynamically (de Zee et al., 2007; Erdemir et al., 2007; Bayoglu et al., 2019). New approaches omit the specification of accurate kinematic data for the spine by forward dynamics simulation of a fully articulated spine (Rupp et al., 2015; Malakoutian et al., 2018; Meszaros-Beller et al., 2023) and including stiffening effects caused by compressive loads (Wang et al., 2020). With strength in dynamic full body approaches, MB models are limited in the study of structural behavior including the load distribution between discs, facet joints, and ligaments as well as the direct prediction of intradiscal pressures (IDP) (Azari et al., 2018). FE analyses, on the other hand, enable a detailed investigation of the structural behavior of the passive ligamentous spine and thus an estimation of internal strains and stresses by discretizing deformable components and contacts (Ayturk and Puttlitz, 2011). Effects of muscle forces and body weight, however, need to be modeled by forces and moments as simplified boundary conditions that do not represent realistic *in vivo* posture or loading conditions, essential to provide a realistic mechanical environment (Favier et al., 2021b; Rajaee et al., 2021; Abbasi-Ghiri et al., 2022).

In particular, clinical problems often exceed the sole capabilities of FE or MB models (Lipphaus et al., 2021), which is why model coupling (Khoddam-Khorasani et al., 2018; Liu et al., 2018; Khoddam-Khorasani et al., 2020; Favier et al., 2021b; Honegger et al., 2021; Kumaran et al., 2021; Panico et al., 2021) is increasingly used. At least two separate spine models using different methods and solvers, are coupled to use results of the other model as complementary input data. Mostly, loading modes are first calculated using an inverse dynamic musculoskeletal MB model and then applied to the passive elements of an implicit FE model for a subsequent detailed structural mechanical analysis (Nispel et al., 2023). That allows the prediction of non-linear internal strains and stresses under combined loading modes that better mimic *in vivo* loads. However, a coupling that leads to valid results exceeds the purely technical challenge of a manual or automated data transfer between two models built in different programs. For results from one model to be used as a valid input to another, both models must be similar, or at best the same, in their mechanical response and morphology. Even if these conditions seem obvious, it is essentially the reason why FE and MB models are coupled: Their approaches to building passive motion segments are inherently different. Liu et al. (2018) described the challenges of adjusting the biomechanical responses of both models under similar loads, arising, for example, from the fact that the intervertebral discs of the MB model were simulated by spherical joints and thus do not allow for deformations, while they were deformable in the FE model. Large movements or high loads reduced the model synchronicity, which required individual corrective measures to be taken (Liu et al., 2018). When using an upstream FE model to initially estimate the nonlinear spine stiffness of the MB model, only simplified load cases are available and the resulting FE motion is the input kinematics for the MB model (Panico et al., 2021). To circumvent this problem, Khoddam-Khorasani et al. (2018) solved a coupled model staggered-iteratively until the FE and MB solutions were convergent. Common to current coupled simulations is that the MB models used use an inverse dynamics approach. The results are thus mainly dependent on accurate kinematic data, which most of the current technologies can neither provide (Meszaros-Beller et al., 2023) nor are available for an *in vivo* equivalent of a MB model.

A hitherto less established way to overcome drawbacks of coupled model simulations are active hybrid FE-MB models. They represent an approach to combining the complementary strengths and limitations of both types of modeling for more predictive models (Lloyd et al., 2019; Knapik et al., 2022). Passive hybrid models provide the inherent combination and interaction of multiple elastic FE and rigid bodies in one model (Stavness et al., 2011). Earlier motivation for passive hybrid spine modeling was the increase of computational efficiency, with advantages for a simplified model structure and increased usability in clinical routine (Deacy et al., 2010; Moramarco et al., 2010; Coombs et al., 2011; Dicko et al., 2015). Likewise, this enabled dynamic solving of complex biomechanical systems with large deformations using explicit FE environments (Shirazi-Adl et al., 2002; Knapik et al., 2012; Rao, 2012). A hybrid model that comprises additional force actuators such as muscles can be referred to as an active hybrid model (Alizadeh et al., 2019). In 2012 Knapik et al. introduced an active hybrid model of the lumbosacral spine (LSS) built in a multi-body dynamic simulation environment to investigate the effects of a total disc replacement at L5/S1 level. Therefore, muscle forces were represented as force vectors driven with electromyographic (EMG) data from a healthy subject. This allowed comparison of stresses in the intervertebral discs and facet joints as well as the range of motions of the individual segments before and after disc replacement. However, their model did not focus on the computational prediction of muscle forces to drive and stabilize the spine. Another active hybrid approach was presented by Rajaee et al. (2021). In their model, the active musculature of a MS model (Arjmand and Shirazi-Adl, 2006a) was integrated into a FE model of the thoracolumbar spine (Shirazi-Adl, 1994) with the aim of fully incorporating the nonlinear stiffness properties of the passive spine. Muscle activities were determined using static optimization algorithm.

In summary, current active hybrid and coupled LSS models have in common that mostly by using established simulation environments, the potentials of the different modeling approaches can only be utilized to a limited extent. Understanding of spinal stability highlights this, as the spinal stabilization system is based on the interaction of three subsystems (Panjabi, 2003; Reeves et al., 2019): the intrinsic stability of the passive spine, the dynamic stability of the spinal muscles surrounding the spine, and the neural control unit that coordinates muscle responses. In more detail, this also includes, for example, the stabilizing influence of the intra-abdominal pressure (IAP) (Hodges et al., 2005), the exact morphology of the facet joints (Khoddam-Khorasani et al., 2018; Knapik et al., 2022), the load sharing (Liu et al., 2018), and the non-linear stiffening of the intervertebral discs under compression (Edwards et al., 1987; Wang et al., 2020). Consequently, an inherent and sufficiently detailed combination of the relevant passive and active spinal structures is necessary, without prescribing accurate kinematics.

Such models, capable of biomechanically investigating the concept of spinal stability under both physiological and pathological conditions (e.g., fusions, injuries, and degenerations), are much needed. Towards this goal, we aim to:

1) Develop a novel forward dynamic active hybrid FE-MB model of the LSS that incorporates the three subsystems involved in spinal stability and moves and balances through a muscle-driven approach.
2) Calibrate and validate the biomechanical model responses of this active hybrid model with literature data measured *in vivo* during various activities.

## 2 Material and methods

**FIGURE 1:**
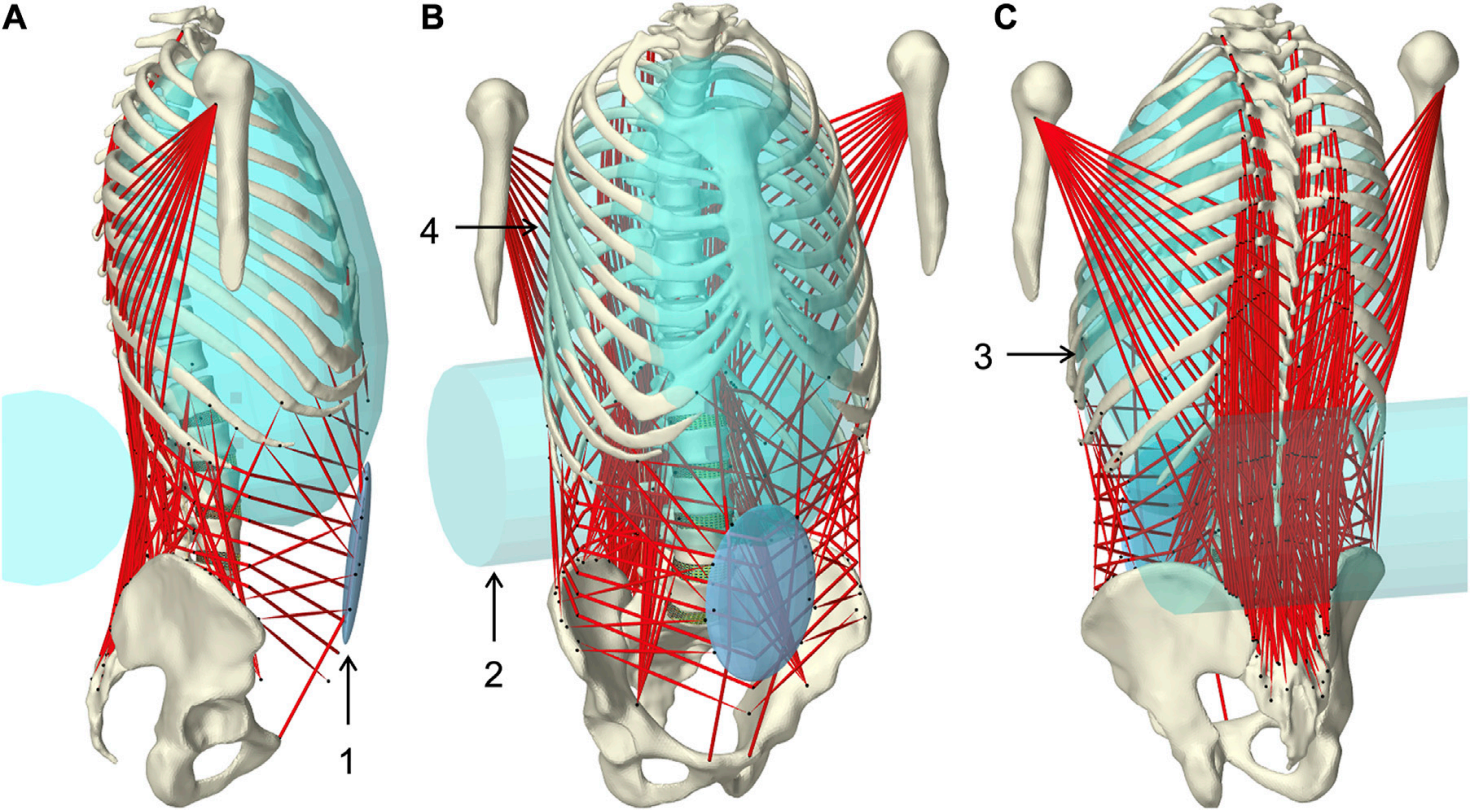
Muscle-driven forward dynamic active hybrid lumbosacral spine model with muscles in red and cyan colored auxiliary rigid bodies (Table 1): 1) Abdominal plate, 2) lumbar wrapping body, 3) left and 4) right thoracic wrapping body. Visualization from three views **(A)** right lateral, **(B)** right front, and **(C)** left rear.

In the following, we cover our approach to building the active hybrid model of the LSS (Figure 1), followed by calibrating the maximum specific muscle tension, calibrating the lumbar segmental rotation contributions of the vertebral target frames, and the systematic testing procedure of the models’ mechanical responses under different load cases (LCs) for validation. The Java-based open-source framework ArtiSynth (www.artisynth.org), a physics simulator that supports the combined simulation of MB and FE models (Lloyd et al., 2012), was used to implement and run the active hybrid model. To perform musculoskeletal geometry modeling and visualize biomedical data in the preprocessing phase we used the software application NMSBuilder (v2.1) (Valente et al., 2017). Downstream evaluations of simulation results were carried out with Matlab (R2020b, MathWorks Inc., US). To encourage open science in spine biomechanics, the model data and procedures developed are described in detail or made freely available.

### 2.1 Passive anatomical model

The anatomy and mechanical properties of the underlying passive hybrid FE-MB LSS model were the same as reported previously (Remus et al., 2021). It consisted of five rigid lumbar vertebrae L1–L5, the rigid sacrum S1, the fiber-reinforced FE intervertebral discs, the facet joints, and the pre-tensioned ligaments. The discs were composed of the quasi-incompressible nucleus pulposus and the surrounding annulus fibrosus with five crisscrossed collagen fiber rings. Hyperelastic material models (Yeoh and Mooney-Rivlin) were used to describe the complex nonlinear stress-strain behavior. The entire passive model is available on GitHub (https://github.com/RemusR9/artisynth_lumbosacralSpineModel) and Zenodo (https://doi.org/10.5281/zenodo.4453702). The anatomical basis was the male Visible Human Project (VHP) (Spitzer et al., 1996) which was adapted in the posture of the LSS (Roussouly et al., 2005). The LSS was extended to include the thorax, humeri, and pelvis. Because the focus was on the LSS, the thoracic region was represented as a single lumped rigid body (Arjmand and Shirazi-Adl, 2006a; Ignasiak et al., 2016), consisting of the vertebrae C7 to T12, the ribs, and the superior segments of the humerus. Thorax and L1 were rigidly connected. With respect to lordosis and a sacral angle of 36.3°, the pelvic alignment was adjusted according to Le Huec et al. (2019) to 45.36° angle of pelvic incidence and 9.06° pelvic tilt to allow sagittal balance in upright standing. The resulting actual tilt of the iliac crest was thus about 2.2° and classifies as an active tight posture balanced between back and abdominal muscles (Schünke et al., 2018). Pelvis and sacrum were rigidly connected because influences of sacroiliac joint on LSS biomechanics for adults are negligible (Le Huec et al., 2019). Upper body segment parameters (Vette et al., 2011) of the male VHP, including center of mass (COM) and moments of inertia, were transformed into the present global coordinate system. The joint centers of the lumbar and thoracic spine serve as points of reference for this. The lumped segment parameters were applied to each rigid model component (Supplementary Material S1). Parameters cranial to L1 were concentrated in COM_UB_ and applied to the thorax. The entire model was symmetrical about the mid-sagittal plane.

### 2.2 Musculoskeletal geometry

**FIGURE 2:**
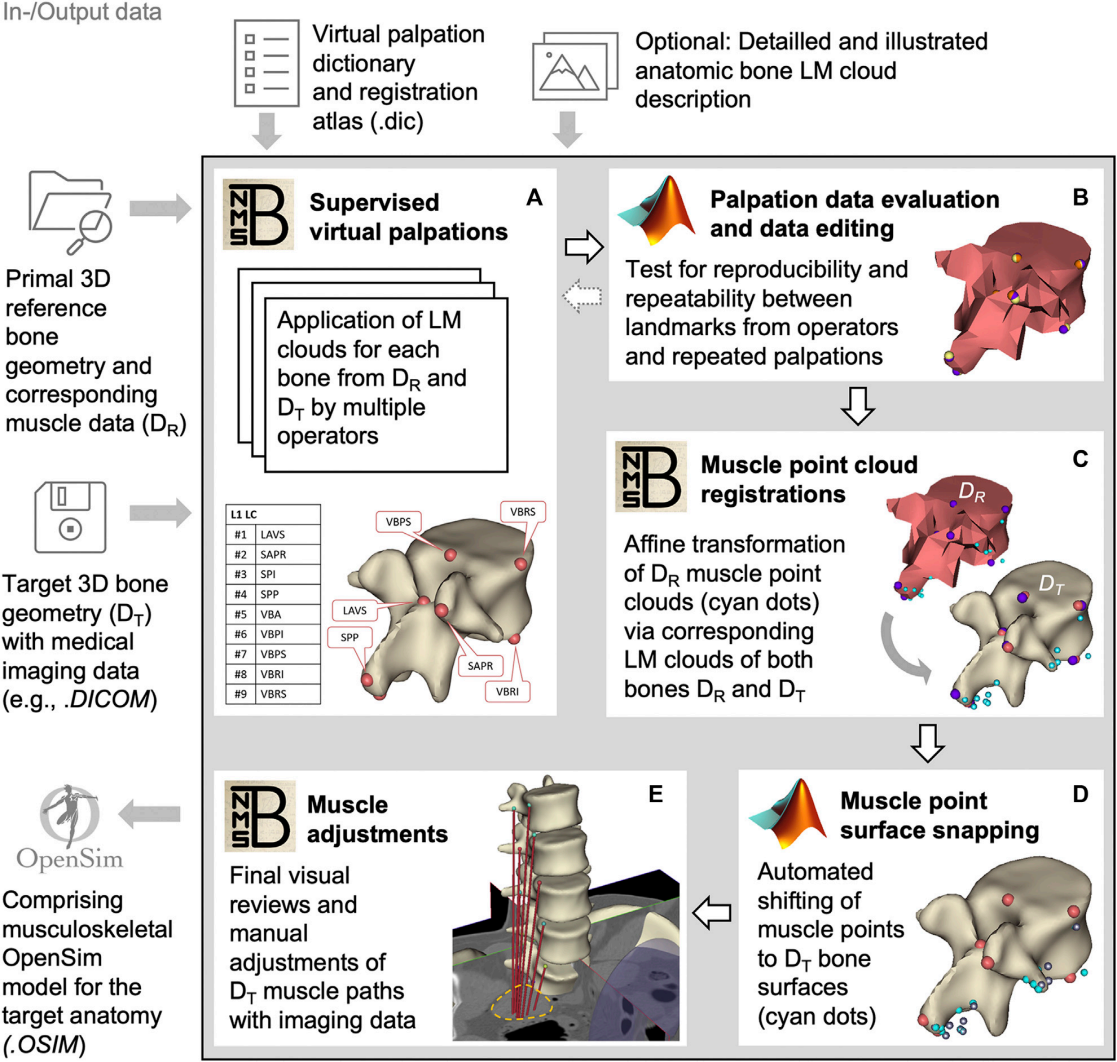
Block diagram of the semi-automated muscle registration process with the relevant in- and output data. The process outlined in the gray box is divided into five general steps **(A–E)**. For each step, the most relevant software, NMSBuilder or Matlab, is indicated. Vertebra L1 serves as an illustration in steps **(A–D)**. The manual adjustment of the muscle paths in e is shown for psoas major.

The active musculoskeletal model extended the passive hybrid FE-MB LSS model to include the muscles of the lower back and abdomen. Our workflow was based on a semi-automated procedure for generating subject-specific musculoskeletal MB models of lower extremities (Ascani et al., 2015; Modenese et al., 2018). We adapted the procedure to efficiently and reproducibly transfer reference model muscle data to a target model. The input data used in the following (Figure 2) are provided in Supplementary Materials S2–S4.

For this, we initially created three skeletal models in NMSBuilder: 1) the modified skeleton of the VHP as the target anatomy (according to the passive anatomical model described in 2.1), plus 2) the OpenSim skeleton “Lumbar_C_238” from Christophy et al. (2012) and 3) the “Twente Spine Model” skeleton by Bayoglu et al. (2017) both as reference models. To describe bone geometries used to register a cloud of muscle points, supervised virtual palpations of anatomical landmarks (LMs) were performed by three operators experienced in back anatomy (Figure 2A). To identify and mark the anatomical characteristics with LMs (Van Sint Jan, 2007), the palpation dictionary (Supplementary Material S2) with anatomical descriptions of the LM positions (created in advance) was available to each operator after a technical introduction. There was the option to use an illustrated documentation of the palpated target anatomy during the initial palpation process (Supplementary Material S3). The registration atlas (Supplementary Material S4) facilitates the workflow in NMSBuilder by providing all LM names per bone in sorted order. All LMs of a bone were aggregated as a LM cloud. To verify repeatability, LM positions were examined for their mean squared distances from the respective mean for each model separately (Figure 2B). A consistent set of anatomical LM clouds was selected for each model for the registration of the reference muscle points (Figure 2C).

**FIGURE 3:**
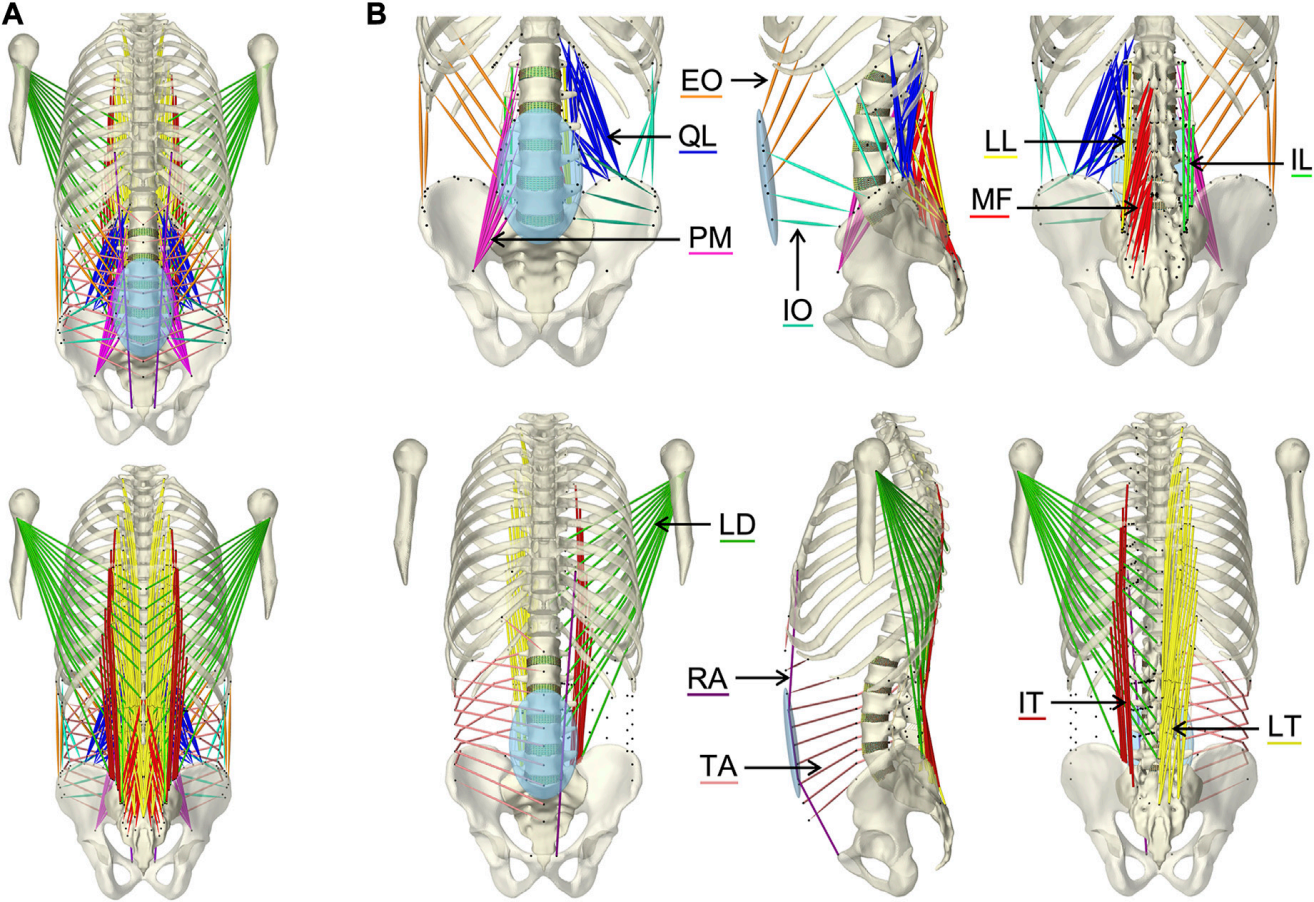
Visualization of the muscles of the active hybrid lumbosacral spine model: Latissimus dorsi (LD), quadratus lumborum (QL), multifidus (MF), obliquus internus abdominis (IO), obliquus externus abdominis (EO), transversus abdominis (TA), psoas major (PM), rectus abdominis (RA), iliocostalis thoracis (IT), iliocostalis lumborum (IL), longissimus thoracius (LT), and longissimus lumborum (LL). **(A)** All muscles differentiated by color in anterior and posterior view. **(B)** One side of each muscle plus the respective muscle points of both muscle sides (black dots) from three views each: anterior, left lateral, and posterior.

The implemented main muscle groups (Figure 3) of the lower back (Christophy et al., 2012) included latissimus dorsi (LD), quadratus lumborum (QL), multifidus (MF), obliquus internus abdominis (IO), obliquus externus abdominis (EO), psoas major (PM), rectus abdominis (RA), iliocostalis thoracis (IT), iliocostalis lumborum (IL), longissimus thoracius (LT), and longissimus lumborum (LL). To complete the abdominal muscles compressing an abdominal cavity (Cresswell et al., 1992; Stokes and Gardner-Morse, 1999; Daggfeldt and Thorstensson, 2003; Essendrop and Schibye, 2004), we included the transversus abdominis (TA) from Bayoglu et al. (2017). Muscles can be divided into functional, one-dimensional fascicles and were modeled in three different forms to best represent muscle paths (de Zee et al., 2007): as a straight line directly connecting insertion and origin point, redirected by means of via-points, and non-linear wrapped around auxiliary bodies. Reference marker clouds consisted of the respective muscle points with reference LM clouds for each bone. Once combined and imported to the target model, an affine transformation was used to register a reference marker cloud on the target model. Raw specific target muscle point clouds were created for each bone. Because anthropometric differences of the bones remained despite the registration, not all muscle points touched the surfaces of the target bones (Figure 2D). Excluding via points, the muscle points were shifted to the bone surface with the least distance using a customized Matlab script (Modenese et al., 2018). All muscles of the right side of the body were generated from the automatically corrected muscle points in NMSBuilder.

Wrapped muscles were redirected by three geometric auxiliary bodies (Table 1; Figure 1). Left and right wrapping body were rigidly attached to the thorax and redirected the long posterior muscles. The lumbar wrapping body was connected to the sacrum via a linked hinge and slider joint. Both joint centers were located in the L5 superior articular process (*x* = −0.02 m, *z* = 0.0345 m). The degrees of freedom of the kinematic joint chain 1) rotation about the joint *y*-axis and 2) translation along the slider joint rotating with the hinge joint, were controlled in extension by the position changes of vertebrae L1 and L2: 1) The lumbar wrapping body was rotated such that the angular sum of the vectors S1-L1 and S1-lumbar wrapping body to the perpendicular on S1 remained constant. 2) Anterior translation with a ratio of 0.54–1, when the *z*-distance between L2 and S1 reduced. In flexion, only the positional change of L4 was used for the sole translational displacement anteriorly along 2) with a ratio of 1.3 to 1.

**TABLE 1:** Geometric description of auxiliary rigid bodies for muscle interactions in reference to the global coordinate system. Dimensions and transformations are given in *x*, *y*, *z* coordinates following the NMSBuilder modeling convention. Visualization of the numbered auxiliary bodies in Figure 1.

#	Rigid body	Geometry	Dimension (m)	Translation (m)	Rotation (deg)
1	Abdominal plate	Ellipsoid	0.008, 0.05, 0.08	0.1345, 0.0, 0.0488	0.0, 7.0, 0.0
2	Lumbar wrapping body	Cylinder	0.3, 0.085	−0.13935, 0.1057	90.0, 0.0, 0.0
3	Left thoracic wrapping body	Ellipsoid	0.1, 0.14, 0.21	0.04287, 0.02059, 0.2788	−4.5, −9.4, 36.4
4	Right thoracic wrapping body	Ellipsoid	0.1, 0.14, 0.21	0.04287, −0.02059, 0.2788	4.5, −9.4, −36.4

For musculoskeletal finalization, the muscle paths and attachment points were manually verified with the VHP image data (Figure 2E) and anatomical descriptions (Hansen et al., 2006; Bogduk, 2012; Adams et al., 2013; Schünke et al., 2018). The generated muscle paths resulted directly from the muscle points transferred to the target anatomy. Minor adjustments were made by relocating muscle points on the bone surfaces, considering muscle wrapping. The final musculoskeletal target model created in NMSBuilder included all bones, the right-sided muscles (Table 2, Supplementary Material S5), and the four auxiliary bodies (Table 1). The right-side musculature comprised 129 (119 without TA) muscle fascicles. For the transfer to ArtiSynth, an OpenSim (Delp et al., 2007) model file (.osim) was exported. The ArtiSynth “OpenSimParser” enabled the import and subsequent integration of the musculoskeletal data. The left-side musculature was mirrored on the sagittal plane.

**TABLE 2:** Overview of the muscle data for the right side of the model.

Muscle group	Fascicle count	Wrapped fascicle count		References
		Lumbar body	Thoracic body	
**QL**	18	0	0	Christophy et al. (2012)
**PM**	11	0	0	
**MF**	25	1	0	
**IT**	8	7	0	
**IL**	4	4	0	
**LT**	21	2	5	
**LL**	5	1	0	
**RA**	1	0	0	
**IO**	6	0	0	
**EO**	6	0	0	
**LD**	14	3	14	
**TA**	10	0	0	Bayoglu et al. (2017)

### 2.3 Musculotendon models

Each muscle fascicle of the 12 implemented muscle groups was described as a tension-only force-generating spring-damper system and modeled via a Hill-type muscle model (Zajac, 1989). Muscle parameters were specified using the “Millard2012AxialMuscle” material, with tendons assumed to have no compliance and non-linear normalized force-length as well as force-velocity curves by Millard et al. (2013). The reference parameters for physiological cross-sectional area (PCSA), fiber to tendon length, sarcomere length, optimal fiber length, pennation angle, and tendon slack length were taken from the respective sources (Christophy et al., 2012; Bayoglu et al., 2017). Multiplying the reference PCSA with the model specific muscle tension *K* (*cf.* section 2.7) gave the maximum isometric muscle force $F_{0_{ref}}^{M}$ . Because the muscle volume is proportional to the body mass *b* and that the muscle-fiber length is proportional to the musculotendon length $l^{MT}$ , the maximum isometric reference muscle force $F_{0_{ref}}^{M}$ was scaled according to Eq. 1 (Correa and Pandy, 2011).

$$F_{0}^{M}=F_{0_{ref}}^{M}\cdot \frac{b}{b_{ref}}\cdot \frac{l_{ref}^{MT}}{l^{MT}}$$ (1)

Musculotendon lengths were extracted from the three fully built musculoskeletal models (*cf.* section 2.2). A sarcomere length of 2.8 µm and a tendon slack length when the muscle is in neutral position was assumed (Christophy et al., 2012). The muscle modeling parameters and muscle attachments used are provided in Supplementary Material S5.

### 2.4 Abdominal kinematic

**FIGURE 4:**
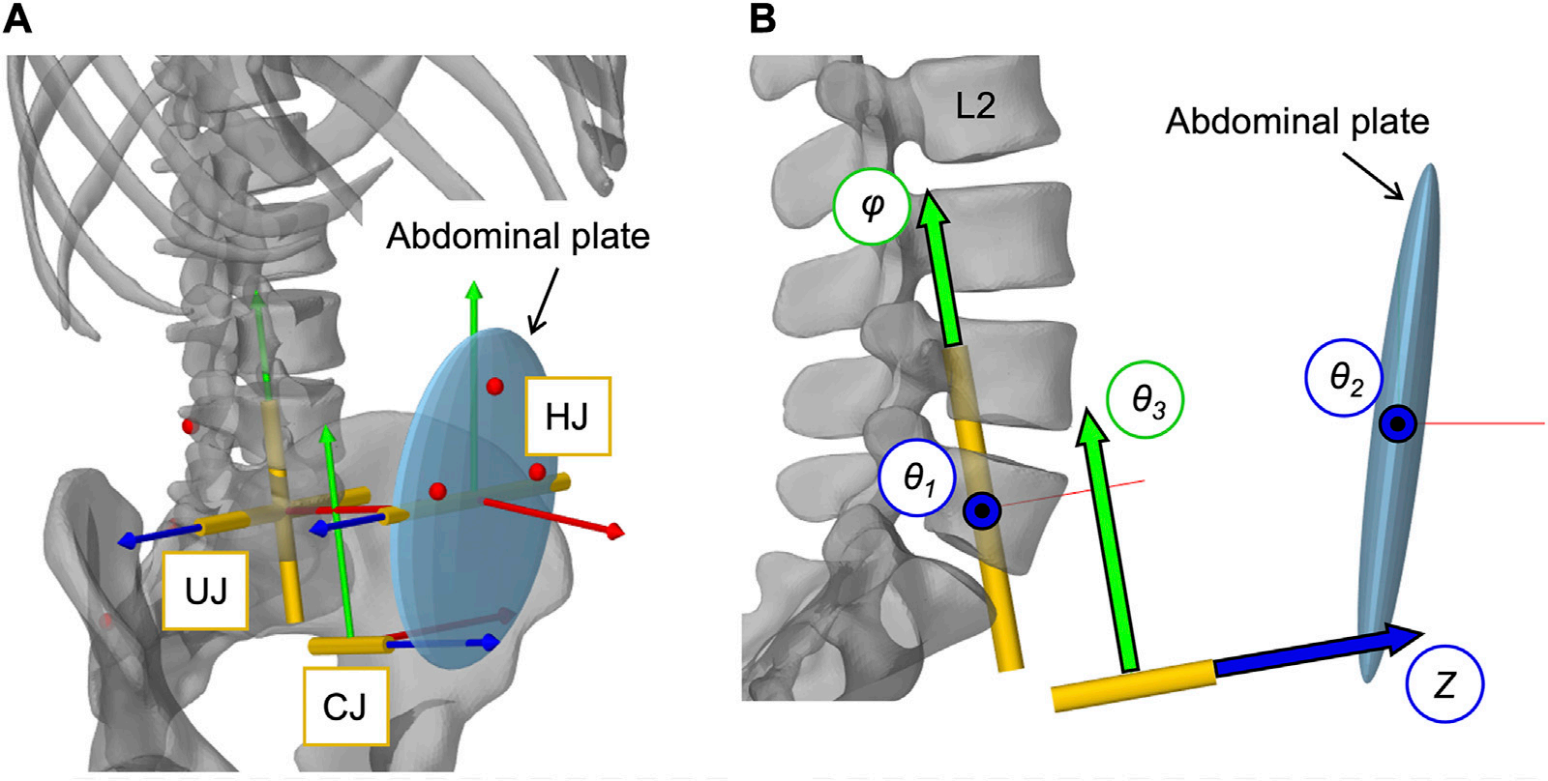
Implementation of the open kinematic chain to control the abdominal plate. **(A)** Visualization of the three joints (HJ, UJ, CJ) with their local coordinate systems connecting the abdominal plate with the sacrum (cf. Table 3). Virtual representatives of the motion capture markers are shown as red dots. **(B)** Lateral view of the three joints with their five controlled coordinates, visualized as arrows with black frames: Rotation angles *θ*_*1*_, *θ*_*2*_, *θ*_*3*_, and *φ* as well as translation distance *Z*.

To define the movements of the abdominal muscles attached to the elliptical abdominal plate (AP), we implemented an open kinematic chain of three linked joints (Figure 4A). The kinematic chain connected AP to sacrum and was composed of three joints: 1) universal joint (UJ), 2) hinge joint (HJ), and 3) cylindrical joint (CJ). Two massless auxiliary bodies in the centers of HJ and CJ (Table 3) were used to connect the joints. The origin of UJ was located in the center of vertebral body L5. The complete kinematic chain was: Sacrum–UJ–Auxiliary body 1–HJ–Auxiliary body 2–CJ–AP. For full kinematic control of the five degrees of freedom, the joint coordinates *θ*_*1*_, *θ*_*2*_, *θ*_*3*_, φ, and *Z,* visualized in Figure 4B, were determined by the angular changes *α*_*j*_ about the three principal axes *j* of the thorax with respect to the pelvis and the constantly held volume of the abdominal cavity. This kinematic correlation was implemented via a polynomial function according to Eq. 2.

$$CC\;\left(\alpha_{j=x,y,z}\right)=p_{1}\alpha_{j}+p_{2}\alpha_{j}^{2}$$ (2)

**TABLE 3:** Abdominal kinematic chain joint settings with polynomial coefficients *p*_*i*_. Given are the global joint centers (*x*, *y*, *z*) and the rotational transformations in respective axis order. Axes after an elementary rotation are denoted with apostrophes. Case differentiations were made for flexion and extension.

Joint	Global joint centers (m)	Joint coordinate system rotations	Controlled joint coordinate CC		Case sensitive polynomial coefficients *p*_*i*_	
					Flexion (*α*_*y*_ > 0)	Extension (*α*_*y*_ < 0)
UJ	0.0080, 0.0, 0.02314	*Rx* = 90.0°, *Rz′* = 10.0°	*θ*_*1*_ *(* *α* *_y_)*	Rotation about the joint *z″*-axis	*p*_*1* _= 0.442, *p*_*2*_ = 0.0116	*p*_*1* _= 0.2437, *p*_*2*_ = 0.0144
			*φ* *(* *α*_*x*_ *+α*_*z*_ *)*	Rotation about the rotated joint *y″*-axis	*p*_*1*_ = 0.4406	
HJ	0.1347, 0.0, 0.0492	*Rx* = 90°	*θ*_*2*_ *(* *α*_*y*_ *)*	Rotation about the joint *z′*-axis	*p*_*1*_ = 0.2509	*p*_*1*_ = 0.4236
CJ	0.0539, 0.0, −0.0308	*Rx* = 90°, *Rz′* = 10°, *Ry″* = 90°	*Z* *(* *V*_*AC*_ = *const*.*)*	Translation along the joint *z″′*-axis	*p*_*1*_ = −0.1663	*p*_*1*_ = −0.4236
			*θ*_*3*_ *(* *α*_*x*_ *)*	Rotation about the rotated joint *z″′*-axis	*p*_*1*_ = 0.815	

**FIGURE 5:**
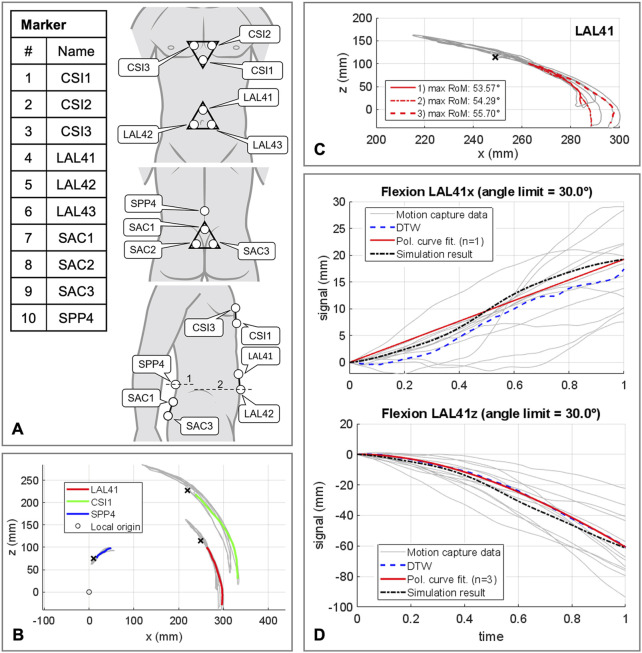
Procedure for determining the polynomial coefficients *p*_*i*_ of the abdominal kinematic chain. **(A)** Marker setup applied to the participants skin. All marker named with a trailing index of 1-3 were fixed to rigid equilateral triangles with respective distances of 60 mm. In lateral view line 1 is crossing the spinous process of L4 and line 2 runs at the level of the upper edge of the iliac crest. **(B)** Sample representation of processed motion capture measurement data for one participant in flexion and extension. Color coded motion curves from neutral posture to flexion for markers LAL41, CSI1, and SPP4. **(C)** Summarization of three motion curves of a participant for the marker LAL41. **(D)** All consulted motion capture curves in sagittal plane in flexion for marker LAL41 with the calculated DTW reference curves as well as the graph of the polynomial functions. Plotted for this purpose are the trajectory components of the same marker at the abdominal plate in the simulation model with optimized *p*_*i*_ (Table 3).

The polynomial coefficients *p*_*i*_ (Table 3) were determined using the procedure visualized in Figure 5 plus downstream optimization by combining *in vivo* measurements and simulations. For this purpose, motion capture recordings (Vicon Vero v2.2 system with 12 cameras) of four healthy, trim men (30.75 ± 1.5 years, BMI 22.67 ± 1.62) were made. Ethic approval was obtained from the Ethics Committee of the Medical Faculty of the Ruhr-University Bochum (23-7801 04/20/23) and written informed consent was obtained from all participants prior to inclusion in the study. Ten reflective markers with a diameter of 14 mm were securely attached to the volunteers’ skin with fixing tape directly or interconnected with a plastic triangle, as shown in Figure 5A. At the same locations virtual marker pendants were added to the simulation model. Starting in an upright position, the participants performed three upper body movements in flexion-extension, axial rotation, and lateral bending. Each to their maximum voluntary range of motion. Participants were instructed to move slowly, at their own pace, and to move primarily out of their lower back. Before starting the measurements, participants undertook at least one practice movement in each direction to ensure smooth recording. During the recordings, attention was paid to ensure that the pelvis tilted little, there was no breathing during the movements, and the trunk muscles were slightly tensed. To process the raw measurement data, individual 3D marker motion curves were extracted starting from upright standing (Figures 5B, C), trimmed based on the maximum thorax range of motion (ROM) (flexion = 30°, extension = −20°, lateral bending = 15° and axial rotation = 10°), and projected to the respective principal motion plane. In order to obtain one valid reference curve for each direction of motion, the individual curves of the marker LAL41 were combined for each coordinate with the modified dynamic time-warping (DTW) method (Wang and Gasser, 1997; Bender and Bergmann, 2012). Lines of best fit with the matching polynomial interpolation degree for each coordinate are listed in Supplementary Material S1 and visualized for flexion in Figure 5D.

Kinematic similarity between AP and *in vivo* data was evaluated by comparing the components of the motion curves of the marker LAL41. To find the ten polynomial coefficients p_i_ (Table 3), that provide the highest kinematic compliance, five multicriteria optimizations (*patternsearch* from the Matlab 2020b optimization toolbox) with global settings were performed. One in each direction of motion independently to determine initial values for p_i_ for the final optimization in all directions combined. Target values to be minimized were the unnormalized distances from modified DTW (Bender and Bergmann, 2012) between both LAL41 coordinate components of polynomial curves and simulated virtual marker trajectories. In all cases the unnormalized distances were summed up unweighted via a cost function.

### 2.5 Intra-abdominal pressure

**FIGURE 6:**
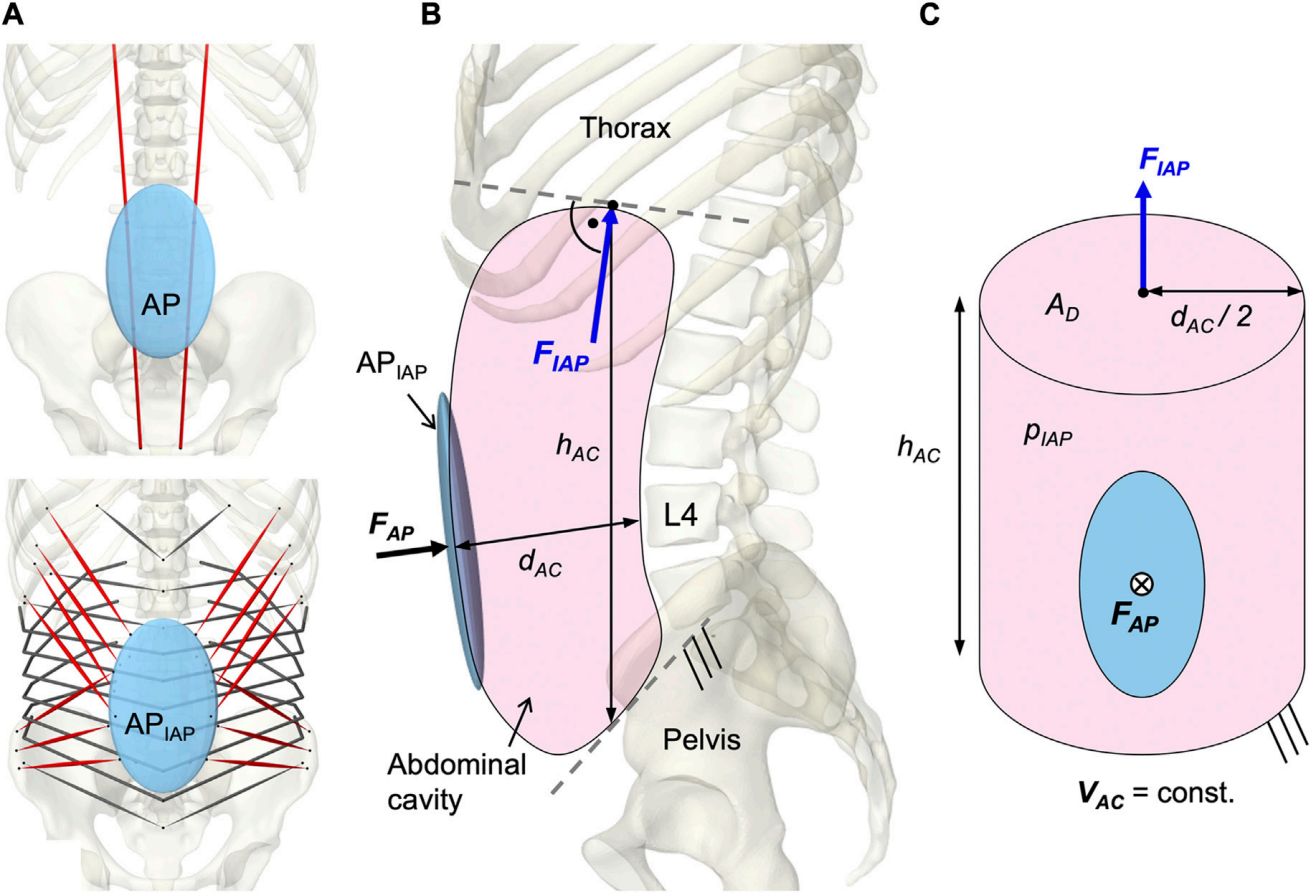
Implementation of the intra-abdominal pressure (IAP). **(A)** Differentiation between the kinematically controlled abdominal plate (AP) and the dynamic active AP (AP_IAP_). The muscle via points of RA were attached to AP. The muscle attachment points of IO, EO, and TA were located on AP_IAP_. Obligatory muscle groups shown in red were IO, EO, and RA. TA was optional and could be deactivated (w/oTA) in the simulation. **(B)** Abdominal cavity with anatomical dimensions, acting forces, and boundary conditions. **(C)** Idealized cylinder with geometric relations between surfaces and forces.

To model a resulting IAP from the abdominal muscle contraction, a second dynamically active and quasi-massless, abdominal plate (AP_IAP_) was integrated at the same location as AP. This was required because only dynamically active bodies calculate forces beyond their motion. As visualized in Figure 6A, EO, IO, and TA were applied to AP_IAP_. RA was redirected via AP without generating a direct influence on the IAP (Essendrop and Schibye, 2004). AP and AP_IAP_ were connected by a “FrameSpring” (Figure 6B), which is a six dimensional spring that generates restoring forces and moments between rigid bodies. Their linear stiffness in F_AP_ direction was set to 90 kN/m to ensure a stable calculation and to limit the relative AP displacement to 1.5 cm at maximum tension of all abdominal muscles. For small relative movements in the other five degrees of freedom, 1 MN/m and 500 Nm/rad were used. Diaphragm and pelvic floor were assumed to be rigid (Stokes et al., 2011). Considering a constant volume (*V*_*AC*_ *= const*.), the abdominal cavity was calculated as a cylinder with height *h*_*AC*_ and diameter *d*_*AC*_ (Figure 6C). The force *F*_*IAP*_ resulting from the IAP was applied to the thorax. Acting cranially, the application point of *F*_*IAP*_ was the center of the diaphragmatic surface *A*_*D*_, 5.1 cm anterior T12. In order to always act perpendicularly on the diaphragm, the force vector changed its direction with the thorax. The abdominal muscles only partially enclosed the simplifying cylinder barrel and were redirected laterally in such a way that the resulting forces were not considered. Under the geometric assumption of an effective muscle entwinement of *c*_*w*_ = 60%, the relation between *F*_*AP*_ and *F*_*IAP*_ is given in Eq. 3.

$$F_{IAP}=\frac{1}{c_{w}}\cdot \frac{d_{AC}}{4\;h_{AC}}F_{AP}=\frac{1}{4}\;\pi d_{AC}^{2}p_{IAP}$$ (3)

An IAP of 4 mmHg (Andersson et al., 1977; Schultz et al., 1982; Nachemson et al., 1986; Mueller et al., 1998) was applied as offset in the unloaded state and the maximum IAP was limited to 200 mmHg (Essendrop, 2003).

### 2.6 Simulation procedure

**FIGURE 7:**
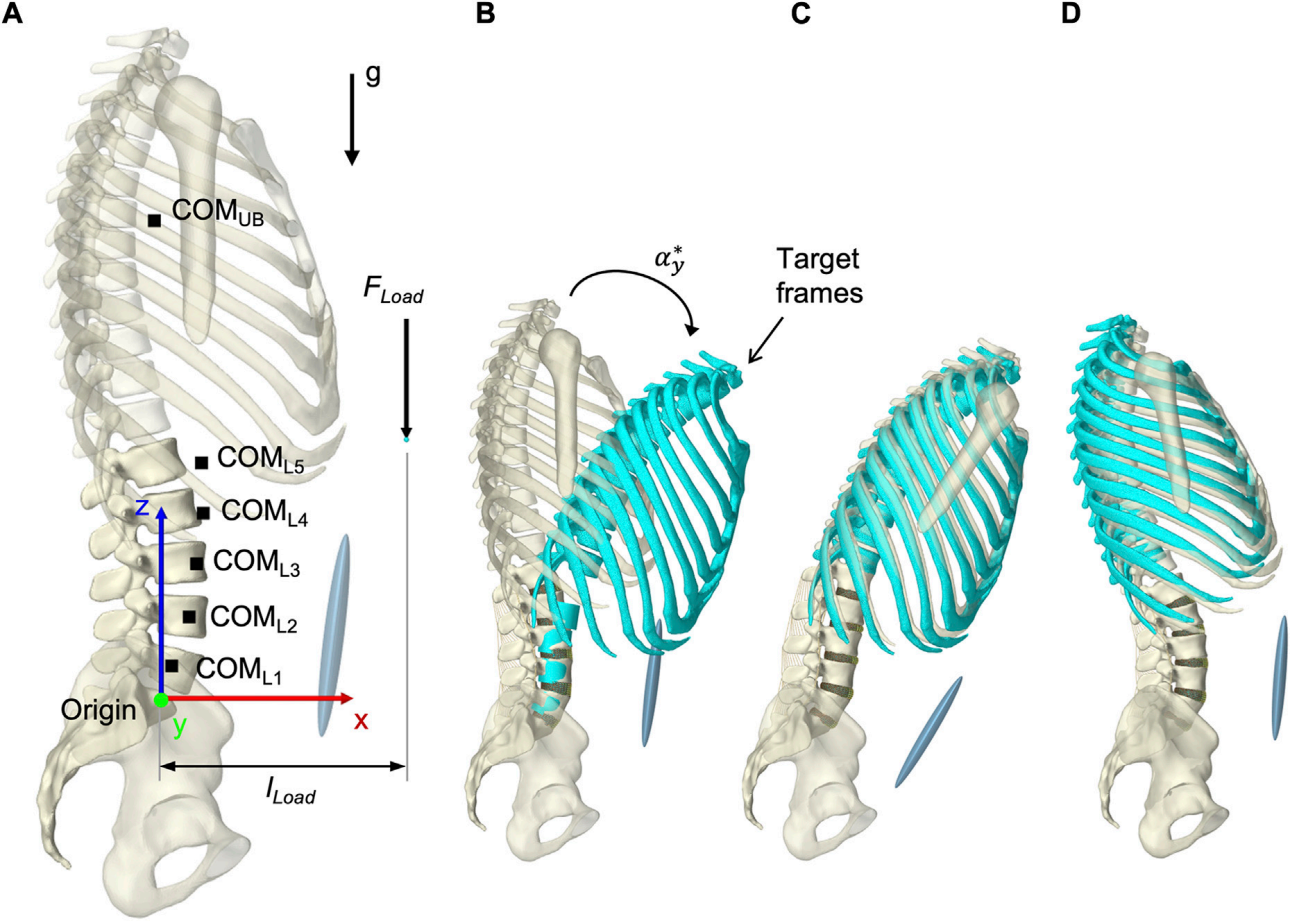
Simulation procedure details. **(A)** Lumped segment parameters, loads applied to the model, and definition of dimensions in upright posture with respect to the origin and the global coordinate system. **(B)** To better represent start and end situations of a simulation, thorax and vertebral body target frames, colored in turquoise, are shown in flexion with $\alpha_{y}^{*}$ = 30° while the model is in the unloaded neutral state. During simulation, all target frames were successively moved so that the motion target term could be continuously minimized by the TC. For the final static condition, the model and the thorax target frame are shown in **(C)** +30° flexion (F30_1_) and **(D)** −10° extension (E10_1_).

To solve the muscle redundancy problem and mimic a physiological muscle recruitment, the tracking controller (TC) integrated in ArtiSynth (Stavness et al., 2010) was used throughout the simulation time. The tracking-based inverse controller provided an optimized solution to the forward dynamics simulation by finding a set of muscle activations at each time step that drives the hybrid FE-MB model along with other constraints through a target movement trajectory (Stavness, 2010; Stavness et al., 2012). Erdemir et al. (2007) classified this approach as “forward dynamics assisted data tracking”. Thus, all postures were generated purely muscle-actuated, without prescribing complete kinematics to the dynamic bones. For the TC motion target term, we specified five target frames for the thorax and the vertebral bodies L2 to L5 (Figure 7B). Its rotational components were used as motion target values. To neglect spinal compression, but constrain displacements in sagittal plane, the translation in *x*-direction was additionally considered for the thorax target frame (Center of rotation: *x* = −0.015 m, *z* = 0.1 m). While the thorax target frame determined the posture of the model, the vertebral body target frames had the subordinate function of reducing oscillations and stabilizing the LSS. Thus, the thorax target values were weighted 15 times higher in the TC cost function. Two additional terms were added to the cost function: The sum of the muscle excitation group activations squared (Stavness et al., 2010) and the rate of muscle excitation changes. The rate of change was added to avoid muscle activation spikes and associated force peaks on the FE discs, which may lead to instabilities. With respect to the thorax target values, the squared excitation term was weighted with 0.667 and the damping term with 0.3×10^−4^. To reduce the number of muscles that were controlled individually by the TC, all muscle exciters were allocated to 12 activation groups for the left and right side, respectively (Table 2).

### 2.7 Calibration of maximum specific muscle tension

Aim of our first calibration was to determine a maximum specific muscle tension *K* by evaluating the maximum isometric back extension that the model could exert. Because *in vivo* measurements demonstrated that the trunk musculature of healthy men generate maximum isometric back-extension torques of 210 Nm in −10° extension and 260 Nm in 10° flexion about the L5/S1 level (Daggfeldt and Thorstensson, 2003) we applied an increasing force equivalent to the thorax at T7: 700 N in extension ( $\alpha_{y}^{*}$ = −10°), 870 N in flexion ( $\alpha_{y}^{*}$ = 10°). The force rotated with the thorax and always acted perpendicularly on it. Gravity was deactivated to mimic the upper body lying on its side. According to Eq. 4 we evaluated equilibrium via the tracking error $∆\alpha_{y}$ as the rotational deviation between the thorax target frame and the thorax.

$$∆\alpha_{y}=|\alpha_{y}^{*}-\alpha_{y}|$$ (4)

Regarding the upright posture after settling (see section 2.9 Validation), $\alpha_{y}^{*}$ defines the rotation of the thorax target frame and $\alpha_{y}$ the predicted rotation of the dynamic thorax in sagittal plane. Reported values for *K* vary between 10 and 100 N/cm^2^ (Daggfeldt and Thorstensson, 2003; Hansen et al., 2006). In models of the lower back, most commonly values of 46 (Bogduk et al., 1992; Stokes and Gardner-Morse, 1995; Christophy et al., 2012), 60 (Arjmand and Shirazi-Adl, 2006a), 90 (Arshad et al., 2016), or 100 N/cm^2^ (Bruno et al., 2015; Bayoglu et al., 2019; Favier et al., 2021a; Lerchl et al., 2022; Malakoutian et al., 2022) were assumed. Thus, we tested values of 46, 73, and 100 N/cm^2^ for *K*, expected $∆\alpha_{y}$ ≤ 1.0°, and that no muscle was fully activated by the TC.

For 46 N/cm^2^, equilibrium could not be established in any posture. At 73 N/cm^2^ equilibrium was achieved only in flexion, with a tracking error of 0.85°. With *K* = 100 N/cm^2^, $∆\alpha_{y}$ was 0.8° in extension and 0.55° in flexion. Furthermore, the model could generate a maximum extension moment of 265 Nm and a maximum flexion moment of 430 Nm for $∆\alpha_{y}$ = 1.0°. This showed a much higher trunk stability in flexion, only considering the tracking error. Because differences in the L4/5 IDP between 73 and 100 N/cm^2^ in flexion were less than 3.0%, we assumed *K* = 100 N/cm^2^ for all muscles. In upright posture, when the abdominal muscles attached to AP_IAP_ without TA were activated to 80% or with TA to 65%, the maximum IAP was generated.

### 2.8 Calibration of segmental target frame rotation contributions

**FIGURE 8:**
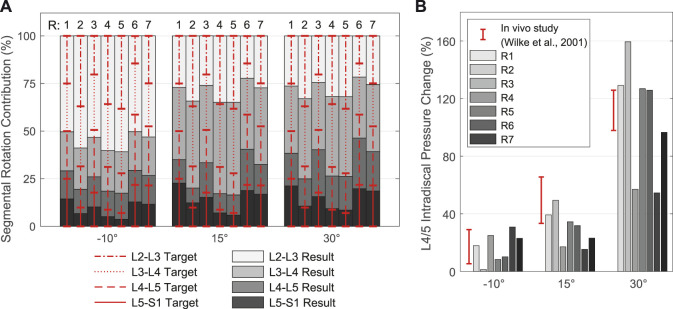
Resulting spinal rhythms for the segmental target frame rotation contributions R1 to R7 used in calibration. All values are given in relation to the respective upright posture (*α*_*y*_ = 0°). **(A)** Comparison of the target frame rotation contributions for L2-L3 to L5-S1 with the resulting IVRs for the three simulated thoracic tilts $\alpha_{y}^{*}$ = −10, +15, and +30°. **(B)** IDP changes for level L4/5 compared with *in vivo* measurements for the same thoracic tilts.

In the second model calibration, segmental rotation contributions for the vertebral target frames (Figure 7B) were determined with respect to their influence on the biomechanical model responses. Consequently, the rotational constraints of the vertebral target frames were part of the optimization problem solved by the TC and influenced the model responses. The deviations between the rotation specifications and the resulting intervertebral rotations (IVRs) of the vertebrae L2 to L5 as well as the calculated IDP L4/5 were used for evaluation. Often referred to as spinal rhythm, *in vivo* studies have shown that the segmental rotation contributions to the overall lumbar motion are level specific and non-linear over the entire motion (Breen et al., 2021). Based on literature sources we tested seven different fixed segmental target frame rotation contributions (Figure 8A):

R1) Proportionally equal contributions of 20% each
R2) Relative contributions averaged over 10°–30° ROM from Wong et al. (2006)
R3) Percentages published by Arjmand and Shirazi-Adl (2006a)
R4) Mean relative segment contributions over 30%–100% L2-S1 ROM in flexion from Breen et al. (2021) with L1-L2 equal to L2-L3
R5) Mean relative segment contributions over 30%–85% L2-S1 ROM in flexion from Breen et al. (2021) with L1-L2 equal to 80% L2-L3
R6) Mean relative segment contributions over 15%–70% L2-S1 ROM in return from Breen et al. (2021) with L1-L2 equal to 80% L2-L3
R7) Relative segmental contributions averaged over 30°–10° flexion to upright from Aiyangar et al. (2015) with L1-L2 equal to 80% L2-L3

In cases where no segmental contribution was measured for L1-L2, we assumed 80%–100% of the contribution of L2-L3, following complete *in vivo* measurements, and adjusted the sum of all five contributions to 100%. Therefore, and because of the rigid connection between T12 and L1 limiting the mobility of L1, we evaluated only the results of L2-S1.

Segmental target frame rotation contributions R1 to R7 and the resulting IVRs are visualized in Figure 8A. The rotational deviations between vertebral target frames and vertebrae were smallest for R4 and R5. Considering all three postures, the root-mean-square errors for R4 and R5 were 9.89% and 9.34%, respectively. Consistent with Arshad et al. (2016), the biomechanical influences of spinal rhythms increased with greater flexion. Directly correlated with this were the IDPs at all levels. Comparison of the percent IDP changes on L4/5 with *in vivo* measurements from Wilke et al. (2001) showed good agreement for all cases except R2, R3, and R6 (Figure 8B). In upright posture, IDP spread was less than 2.8% and IVR deviations were below 0.3°. The choice of segmental contribution was almost irrelevant in phases of low thoracic tilt (−5° < $\alpha_{y}^{*}$ < 5°). Similar to the experimental setup of Breen et al. (2021), the pelvis in our model was stationary. Furthermore, because segmental target frame rotation contributions R4 and R5 provided the most suitable boundary conditions, we used R5 with the lower proportion of L1-L2 for model validation: 23.4% at L1-L2, 29.3% at L2-L3, 25.9% at L3-L4, 16.0% at L4-L5, and 5.4% at L5-S1.

### 2.9 Validation

For validation, the calculated biomechanical model responses of 13 LCs (Table 4) were compared with *in vivo* studies (Nachemson, 1965; Schultz et al., 1982; Sato et al., 1999; Wilke et al., 2001; Arjmand and Shirazi-Adl, 2006a; Takahashi et al., 2006; Wong et al., 2006). These LCs were selected due to the availability of the L3/4 or L4/5 IDP measurements. The LCs comprised sagittal postures of the thorax target frame graded in 10° in the range −10° ≤ $\alpha_{y}^{*}$ ≤ 30° (Figures 7C, D), each with or without the external load *F*_*Load*_. For example, when compared to the measurements from Wilke et al. (2001), the external load approximated holding a 20 kg crate in both hands with arms bent or extended in front of the upper body. In Takahashi et al. (2006) 5 kg were attached to both wrists of the vertically hanging outstretched arms. The force *F*_*Load*_, always acting vertically, moved with respect to the shoulders and their distance from the origin was *l*_*Load*_ (Figure 7A). Dead weight displacements of the arms were neglected. Origin and sternum had a distance of 0.133 m in neutral posture. Biomechanical model outputs used for validation were the IDPs of the three caudal lumbosacral levels, the predicted muscle forces, and the IAP. Each LC was simulated with muscle group TA (w/TA) and without TA (w/oTA). This is due to the combination of two independent data sets for the abdominal muscles and the verification of the sensitivity of the biomechanical model responses.

**TABLE 4:** Experimental plan of the model validation. LCs were taken from *in vivo* studies. *l*_*Load*_ is specified for the given posture in the respective static state.

LC	Thorax posture [ $\alpha_{y}^{*}$ ]	*F*_*Load*_ (N)	*l*_*Load*_ (cm)	References	Comparison responses		
					IDP	IAP	EMG
**N0**_**1**_	Upright [0°]	-	-	Schultz et al. (1982), Nachemson et al. (1986), Sato et al. (1999), Wilke et al. (2001), Arjmand and Shirazi-Adl (2006a), Takahashi et al. (2006)	x	x	x
**N0**_**2**_		80	62	Schultz et al. (1982), Nachemson et al. (1986)	x	x	x
**N0**_**3**_		100	4	Mueller et al. (1998), Arjmand and Shirazi-Adl (2006a), Takahashi et al. (2006)	x	x	x
**N0**_**4**_		200	19	Wilke et al. (2001)	x		
**N0**_**5**_			44		x		
**F10**_**1**_	Flexion [10°]	-	-	Sato et al. (1999), Takahashi et al. (2006)	x		x
**F10**_**2**_		100	10.5	Takahashi et al. (2006)	x		x
**F20**_**1**_	Flexion [20°]	-	-	Sato et al. (1999), Takahashi et al. (2006)	x		x
**F20**_**2**_		100	16.6	Takahashi et al. (2006)	x		x
**F30**_**1**_	Flexion [30°]	-	-	Sato et al. (1999), Arjmand and Shirazi-Adl (2006a), Takahashi et al. (2006)	x		x
**F30**_**2**_		80	70	Schultz et al. (1982), Nachemson et al. (1986)	x	x	x
**F30**_**3**_		100	22.8	Schultz et al. (1982), Arjmand and Shirazi-Adl (2006a), Takahashi et al. (2006)	x		x
**E10**_**1**_	Extension [-10°]	-	-	Sato et al. (1999), Wilke et al. (2001), Wong et al. (2006)	x		

All simulations started in a neutral state without gravity and without external forces with the LSS and thorax in upright position (Figure 7A). To increase computational stability, the resting muscle tone was set to 0.1%, which affected IDPs by less than 1% compared to no muscle tone. The sacrum was stationary, maximum step size was set to 0.01 s, and a first order backward integrator (“ConstrainedBackwardEuler”) was used. Within the first simulation steps, the gravity was ramped up to 9.81 m/s^2^ in the negative *z*-direction. This was followed by a settling of the model, done by a thorax target frame rotation of $\alpha_{y}^{*}$ = 2° and the return to the upright posture. This small reversible movement of all dynamic bones established an energetically favorable condition with minimal IDPs between passive system stiffness, dead weight, and muscle forces. We defined the resulting orientation of the vertebrae as the stable, upright reference posture (*α*_*y*_ = 0°). Due to the rigid connection of thorax and L1, *α*_*y*_ also represented the absolute rotation of L1. Subsequently, the poses were set by moving the target frames steadily, optionally followed by an increase of *F*_*Load*_ in form of a smoothstep function. In Supplementary Material S6, the simulation procedure is visualized as an example for LC F30_3_. Results were evaluated when the entire model reached a static state (equilibrium).

## 3 Results

**FIGURE 9:**
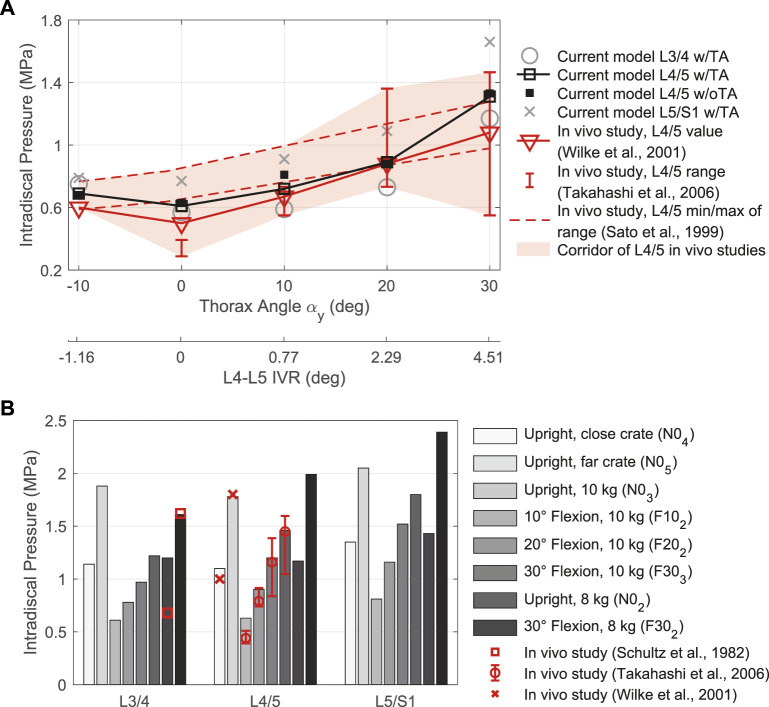
Comparison between IDP results for intervertebral discs L3/4, L4/5, and L5/S1 w/TA with *in vivo* literature data. **(A)** LCs without additional loads in different sagittal postures. Given are the thorax target angles *α*_*y*_ and the predicted L4-L5 IVRs to compare with measurements by Sato et al. (1999). **(B)** LCs with external load (*F*_*Load*_ ≠ 0 N) in different postures.

The resultant lumbar lordosis of the musculoskeletal biomechanical model in upright position (N0_1_) measured from the superior endplate of L1 to the superior endplate of S1 was 47.5°. The IVRs between neutral vertebral orientation and the upright reference posture were from L1 to L5: 4.82, 1.88, −2.71, −3.37, and 3.12°. For all LCs, $∆\alpha_{y}$ was less than 0.22°. The estimated IDPs of the three caudal levels in different postures without additional load are shown in Figure 9A and with different *F*_*Load*_ in Figure 9B. The load on the lumbar spine was lowest in N0_1_ and a minimum pressure was predicted at level L3/4 with 0.59 MPa. From this, the resulting IDPs for L4/5 and L5/S1 increased approximately linearly in flexion up to 20°. Above that, the pressure response was no longer linear. The IDP increased caudally per level. A maximum IDP of 2.39 MPa occurred at level L5/S1 in 30° flexion holding 8 kg with outstretched arms (F30_2_). Comparing the absolute IDP values w/TA as well as their relative changes, there is a high agreement with the *in vivo* measurements (Schultz et al., 1982; Sato et al., 1999; Wilke et al., 2001; Takahashi et al., 2006), shown in Figure 9. However, *in vivo* values for upright standing without external load (Takahashi et al., 2006) and with 8 kg in outstretched arms (N0_2_) (Schultz et al., 1982) are about half lower than simulated. This was not the case for the data of Wilke et al. (2001) and Sato et al. (1999) as well as for all pressure measurements in sagittal deflection. Except for 10° flexion, predicted IDP deviations were on average lower than ±4.4% for the three caudal levels in LCs w/oTA compared to LCs w/TA. Least changes with maximum −1.5% resulted for all LCs in 30° flexion. For N0_1_, the IDPs w/oTA were increased in caudal direction by 3.7, 3.3, and 2.6%. Mean deviations for F10_1_ and F10_2_ were 19.1% and 6%, respectively.

**FIGURE 10:**
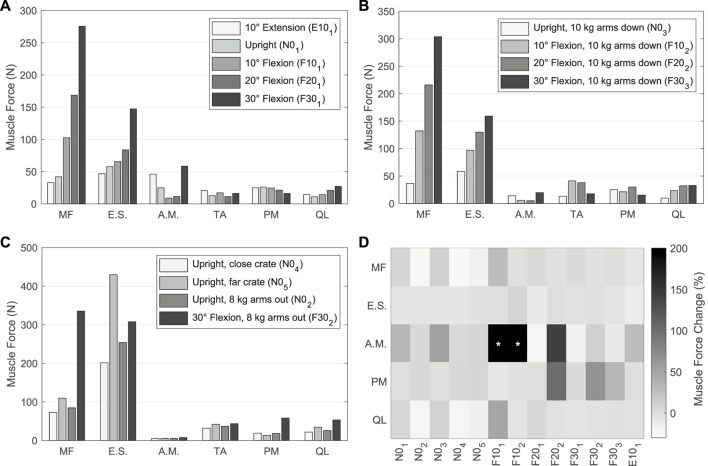
**(A–C)** Predicted muscle forces of the left side of the model w/TA. Forces of IT, IL, LT, and LL are combined into erector spinae (E.S.), and IO, EO, and RA are combined into abdominal muscles (A.M.). **(A)** Sagittal postures without additional loads. **(B)** LCs in which 10 kg were carried with vertically hanging arms. **(C)** Comparison of LCs with different lifting activities. **(D)** Muscle force changes w/oTA compared to values from **(A–C)** w/TA. Values with * exceed a change of 200%.

The muscle forces determined by the TC to drive and stabilize the model are visualized in Figures 10A–C for the validation LCs w/TA from Table 4. For better comparability with *in vivo* literature data (Schultz et al., 1982; Arjmand and Shirazi-Adl, 2006a; Takahashi et al., 2006), the paraspinal muscle forces excluding MF were summarized as erector spinae (E.S.) and abdominal muscles (A.M.) excluding TA. MF and TA are visualized separately. The sum of all muscle forces in N0_1_ including TA was 351 N. In 10, 20, and 30° flexion (F10_1_, F20_1_, and F30_1_) the sums were 468, 636, and 1084 N, respectively, and 374 N in E10_1_. The resulting muscle activation patterns were individual for each LC. That the highest back muscle forces occurred when the thorax was flexed 30° and 8 kg was held in both hands with outstretched arms (F30_2_) is consistent with the measurements of Schultz et al. (1982). Similarly, the estimated force of the abdominal muscles decreased with increasing flexion and *F*_*Load*_. Arjmand and Shirazi-Adl (2006a) also found *in vivo* an increased activity of E.S. and MF in flexion. Correlating with higher weight in the hands, both their activity increased. The increase in E.S. activity in flexion with and without a load of 10 kg measured *in vivo* (Takahashi et al., 2006) correlates closely with our calculated force changes (Figures 10A, B). Consistent with this *in vivo* study, additional loads did not induce significant changes in the predicted abdominal muscle activity. High agreement was also seen for MF inactivity in upright position: Holding a light to heavy weight (N0_2_ - N0_5_) barely led to force changes in the model as well as changes in muscle activity based on EMG measurements by Arjmand and Shirazi-Adl (2006a). To keep the upper body upright, only the muscle groups of the E.S. were activated to a greater extent. In general, in agreement with EMG measurements (Arjmand and Shirazi-Adl, 2006a), Figures 10A, B show a decrease in abdominal muscle activity in flexion. In contrast, Takahashi et al. (2006) measured *in vivo* an up to 2.7-fold increase of RA activity in 20° flexion without additional load. In our model, RA was only activated to a greater extent in maximum flexion (F30_1_) and generated a force that was almost 3.5 times higher than in upright posture. In extension (E10_1_), RA generated twice the force of the upright posture. The force variations of PM and QL between LCs were small, but in relation to N0_1_ maximal for F30_2_.

For comparison of the calculated muscle forces of all LCs w/oTA, the percentage changes to w/TA are visualized in Figure 10D. Generally, the least changes were seen for E.S. and the largest for A.M. When holding heavy loads in upright position (N0_2_, N0_4_, N0_5_), MF and QL exerted forces reduced by up to 24%. Other muscle forces as well as the IDPs were approximately constant. For the upright position without heavy loads (N0_1_, N0_3_), forces of A.M. were increased by up to 50% and QL as well as MF by up to 10%. This had no effect on the IDPs but led to IVR deviations of up to 0.15°. In case of 10° flexion, A.M. were increased primarily due to EO by 400% and 500% for F10_1_ and F10_2_, respectively. In F10_1_, the forces for MF and QL w/oTA were also increased by 28% and 49%. This resulted in a 16% higher IDP for L4/5 (Figure 9A). In flexion ≥20° and extension, the predictions w/oTA resulted in no relevant force changes for MF, E.S., and QL (−2.6% ± 2.8%). Only A.M and PM showed correlative changes in their forces. Effects on IVRs were smaller than 0.2°. Changes for the predicted IDPs were <1.8% for flexions greater than 10° and <3.8% in the upright position.

**FIGURE 11:**
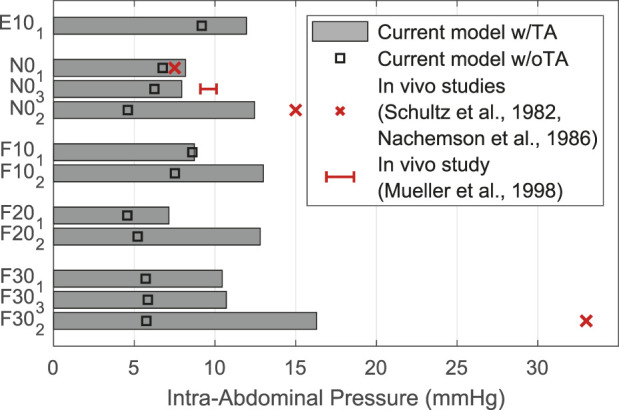
Comparison of predicted IAP w/TA and w/oTA with literature data.

The IAPs calculated by our model are shown in comparison with *in vivo* measured values (Schultz et al., 1982; Nachemson et al., 1986; Mueller et al., 1998) in Figure 11. Highest IAP with 16.30 mmHg and lowest IAP with 7.15 mmHg occurred in F30_2_ and F20_1_. The calculated pressure values w/TA for the upright posture demonstrated a high agreement with measurements of Schultz et al. (1982) and Mueller et al. (1998). w/oTA the IAP was reduced by up to 65%. For F30_2_, the calculated IAP was half as high as measured by Schultz et al. (1982).

## 4 Discussion

This paper presents the modeling procedure, calibration, and validation of a novel muscle-driven active hybrid FE-MB model of the LSS. The modeling procedure included the extension of our previously validated passive hybrid LSS model (Remus et al., 2021) with trunk muscles and auxiliary bodies for muscle attachments and wrapping. Morphological muscle data were integrated via virtual palpation as part of a semi-automated registration procedure. Redundant muscle activations were predicted via a forward dynamics assisted data tracking algorithm to move and balance the LSS in different postures. The auxiliary bodies comprised an AP whose kinematics were optimized using motion capture data of the torso performed in this study. Assuming an incompressible abdominal cavity, the IAP was directly calculated from the posteriorly acting forces of the abdominal muscles on the AP. The aim of the two calibrations in this study was to obtain spinal stability. For this purpose, a specific muscle tension was first determined, followed by the determination of segmental rotation contributions for the vertebral target frames as an optimization criterion. To validate the forward dynamic model, 13 sagittally symmetric LCs from −10° extension to +30° flexion with and without different loads up to 20 kg held with both hands were simulated. Biomechanical model responses used for validation with *in vivo* literature data comprised IDP at L3/4 to L5/S1, IAP, and muscle activation patterns.

### 4.1 Active hybrid lumbosacral spine model

The passive LSS without surrounding active musculature is an unstable system that tends to buckle even under low vertical loading (Patwardhan et al., 1999). The co-contraction of abdominal and lumbar muscle groups combined with the activation of the diaphragm and the pressure that builds up provides an mechanism for stabilization (Hodges et al., 2005; Kuo et al., 2021). Spinal instability, clinically defined as loss of the spine’s ability to maintain its displacement patterns during physiological loads, is an important cause of low back pain (Panjabi, 2003). Destabilizing factors may include force reducing and response degrading dysfunction of MF and TA (Kuo et al., 2021). Persistently increased abdominal muscle tension, in turn, leads to abdominal hypertension (Tayebi et al., 2021) that can lead to health problems such as pain or organ deterioration (van Ramshorst et al., 2011). The physiological interplay of all components is therefore of particular relevance for spinal stability. Within the scope of our musculoskeletal modeling, however, respective interactions in the lower trunk could often only be represented in a simplified way, which is discussed in the following.

The active model built consisted of a trunk musculature in addition to the passive FE-MB LSS. Due to the unavoidable combination of data for anatomy and materials as well as calibration and validation, our model represented an average healthy and middle-aged man without individual characteristics. Consequently, patient-specific conclusions were limited. However, to achieve better comparability of results and to benefit from the wide availability of data, dimensions and masses were based on the Male VHP. For the musculature, we combined morphology and material reference data from two independent sources which thus included the relevant parts of the autochthonous back muscles as well as the abdominal muscles (Table 2). Reason for the combination was our consideration of the IAP (Essendrop and Schibye, 2004) and the omitted TA in the primarily used OpenSim model of Christophy et al. (2012). Limitations of the second data set by Bayoglu et al. (2017) were the fusion of L5 with the sacrum, whereby the authors could not exclude alterations of the measured muscle groups. Because intertransversarii and interspinalis muscles are mainly considered as proprioceptive sensors rather than force actuators (de Zee et al., 2007), we did not supplement them with additional reference data. In addition, compared to other recent models (Lerchl et al., 2022), fascicles not included in the model of Christophy et al., such as MF attachments to the thoracic spine, were not manually added. To integrate the muscle morphologies into our model, a published and established semi-automated codified registration procedure (Ascani et al., 2015; Modenese et al., 2018) was modified and utilized. A challenge in our case was the considerably higher number of muscle groups and fascicles as well as their often non-linear course. Likewise, the degree of morphological detail of the reference bones was different. The sacrum of the OpenSim model, for example, had a very low level of detail, which is why the repeated palpations showed high local deviations. Absolute highest LM variations were found for the largest element to be palpated, the thorax at angulus costae of the 7th and 9th rib and the 7th and 11th corpus costae (see palpation dictionary in Supplementary Material S2). LMs for vertebrae and pelvis could be determined for all data with the highest repeatability. In general, depending on the 3D bone geometry quality and morphology, LMs could be determined with varying accuracy. Compared to the VHP image data imported into NMSBuilder, the paths of the QL fascicles required the most manual correction. After registration, these passed almost completely through the transverse cross-section of the E.S. at level L4. Little to no correction was required for the remaining muscle groups. Overall, the use of the adapted semi-automated registration procedure (Figure 2) had proven to be reliable and efficient. Furthermore, the workflow may become relevant for easy modification of the musculature or for transferring the muscle morphology used here to deviating bone geometries. Also, patient-specific musculoskeletal models can be generated in this way based on one reference model (Modenese et al., 2018).

Four auxiliary rigid bodies were integrated into the model for redirecting or attaching muscle fascicles (Table 1). Of these, the lumbar wrapping body and the AP were kinematically controlled via open kinematic chains. Advantage of using linked joints were the inherent kinematic determinacy with easy manipulability of the chain as well as using scaling factors during investigations and optimizations. The kinematics of the lumbar wrapping body were implemented according to the smaller cylinder integrated in the OpenSim model so that the lumbosacral muscles were kept near the lordotic spine in extension. We did not integrate an equivalent of the larger of the two lumbar wrapping cylinders into the presented model. The smaller cylinder redirected all muscle fascicles for the LCs considered.

Consistent with the state of the art in MB models, the AP replaced all ligamentous structures of the central abdominal wall (Schünke et al., 2018) and defined the anterior muscle attachment points and abdominal muscle movements (de Zee et al., 2007; Christophy et al., 2012; Malakoutian et al., 2018; Liu et al., 2019b). Kinematics optimization of the AP was based on motion capture measurements performed only on a small group of young men. It must therefore be assumed that no universally valid abdominal kinematics was implemented. However, the interindividual high concordance of the measured marker trajectories suggested to us a suitable approximation for this target group. Not all recorded marker movements of our *in vivo* study were used for the presented optimization. Investigations by us, not described further here, showed that consideration of further measured values did not add value to the derivation of abdominal kinematics. Other measured values included the pose of the abdominally applied triangle and the movements of marker SPP4 (Figure 5). Their consideration can be useful, for example, in more complex movements beyond the three main planes or in pathologies. A limitation might be, however, that we based abdominal kinematics solely on the position and orientation of the pelvis and the thorax. As already realized via SPP4, further vertebral body displacements should be considered in the future and thus the absolute distance between lumbar spine and abdominal wall should be taken into account. In addition, it should be noted that abdominal and upper body movements can be measured easily and noninvasively using markers, but both palpation of bony LMs and displacement of the skin can lead to erroneous results.

To consider IAP in simulation models of the lower back, various approaches have been implemented (Han et al., 2012; Arshad et al., 2017; Malakoutian et al., 2018; Liu et al., 2019b). The use of a kinematic chain with two abdominal plates to directly utilize compressively acting abdominal muscle forces from IO, EO, and TA represented a new approach. Since AP and AP_IAP_ had the same geometry and were in the same pose in the model, we counted only AP for the auxiliary bodies. The mechanical effect of the IAP was modeled as a cranially aligned force acting perpendicularly on the diaphragm that rotated with the thorax. To convert between the total force acting on the AP_IAP_ and the IAP, we assumed a constant volume of the abdominal cavity idealized as a cylinder. Both the upper body posture and the correlating AP position therefore had an influence on the IAP. The computed volume change via the IAP thus affected the stability of the system and was part of the overall muscle activation problem. The entwinement factor implemented for scaling was based on the geometrical assumption and the redirection of the TA fascicles by via points. Resulting compression forces acting on the via points were not considered. This resulted in considering only the part of the muscle forces acting perpendicularly on the AP_IAP_. Especially because the factor c_w_ is variable, its variation should be systematically investigated in further studies. Instead of the direct muscle force measurement, the implementation of a fully analytical approach is alternatively conceivable, as in a fundamental work (McGill and Norman, 1987). For the estimation of the IAP, the dynamic AP_IAP_ could be omitted, and redirections of TA would become irrelevant. Only the activity of the muscles of the abdominal wall and the present geometric conditions would be input variables. For a discussion of the difficulties involved, we refer the reader to the work of McGill and Norman (1987). Another limitation of our approach to be considered were the equidistant distances of the abdominal muscles in all postures as a result of the anterior attachments to the rigid AP_IAP_. Potential effects on pressure as well as activation resulted in flexion could not be assessed in more detail at this point. Thus, contraction of the trunk muscles leading to deformations of the abdominal cavity and the associated elevation of the anterior abdominal wall (Todros et al., 2020) could not be studied either. In addition, we neglected the pressure-increasing influences on the abdominal cavity resulting from diaphragm contraction, which may contribute to spinal stabilization (Hodges et al., 2001) as well as the influence of breathing to the control of abdominal muscles (Hodges et al., 2015).

The hybrid modeling approach included five fibre-reinforced FE discs and ten FE inferior articular facets in addition to the rigid bones, muscles, and ligaments. Compared to MB models, this led to several downsides. The modeling effort was considerably increased and is close to FE models (Affolter et al., 2020; Pickering et al., 2021). Manual segmentation and meshing were required, which, along with the level of intervertebral disc detail, is currently a bottleneck for anatomical modification. With the initial aim of directly calculating the IDP and predicting the complex non-linear kinematic responses of the LSS, we conducted a sensitivity study (Remus et al., 2021) to reduce the complexity of the disc. More detailed analysis of the discs is possible, but would require a finer mesh and more parameters, increasing the complexity of the model. The use of FE meshes for discs in active hybrid models could therefore allow the influence of motion and loading on disc degeneration to be studied and, for example, fatigue damage to be predicted (Subramani et al., 2020). Partial or complete replacement of rigid vertebrae with FE bodies is also possible. In the future, this may allow the investigation of structural-mechanical issues under *in vivo* similar loads, for example, to study bone adaptations due to mechanical stimuli (Smit et al., 1997; Lipphaus and Witzel, 2019; Favier et al., 2021b), and the osseous integration of spinal cages (Knapik et al., 2012; Malakoutian et al., 2015; Abbasi-Ghiri et al., 2022). With regard to the already integrated FE bodies and contacts, the computing time has to be mentioned. Using a desktop PC with Intel i7-10700K @ 3.80 GHz, 32 GB Ram and 1 TB SSD running Windows 11 Pro 64-bit, LC F30_3_ took an average of 30 min to calculate. Comparable load cases are calculated in ArtiSynth in several seconds by a purely forward dynamics, muscle-driven MB model (Malakoutian et al., 2018).

### 4.2 Calibration

We calibrated the maximum specific muscle tension and segmental target frame rotation contributions because corresponding literature data from experiments and simulations showed wide variations. The iterative procedure within the calibration, required to ensure consistency of results, was not discussed in this paper. Each simulation started in the same neutral state without external forces, but with ligaments pre-tensioned and the TC activated. This was the state in which the model was geometrically built. During the first simulation steps, gravity was ramped, followed by the settling phase in which the bones were able to move into an energetically favorable state. This was necessary because due to the forward dynamics method (Pandy, 2001) bone poses were not kinematically controlled and the upright stable is unequal to the initial neutral state. In contrast to inverse dynamic models (Christophy et al., 2012; Ignasiak et al., 2016; Khoddam-Khorasani et al., 2018; Liu et al., 2018; Favier et al., 2021a; Rajaee et al., 2021; Lerchl et al., 2022), the kinematic inputs were not complete and only used to move the five target frames (Figure 7B). The model dynamics described how the components advanced in time from one state to another. The exact motions of the active hybrid model, which include the IVRs, were not known in advance. By controlling the muscles using the TC, deviations between all target frames and respective bones were minimized for the selected coordinates. For the four vertebral target frames, these were only their rotations in sagittal plane. Resulting muscular forces directly moved the entire spine into various postures and provided spinal stability (muscle-driven approach).

All LCs studied could be generated by the postural specification of the thorax target frame alone. However, as a sole boundary condition, we found that for some LCs with *α*_*y*_ ≥ 20, no stable state for the lumbar vertebrae L2-L5 could be obtained by the TC. As a result, vertebral oscillations were not reduced by the muscles, which in the case of LC F30_2_ resulted in inverted finite elements due to excessive distortions of the discs. Consequently, the specifications of the vertebral target frame rotations primarily served to stabilize the spine with the premise of keeping the influence on the model dynamics minimal (low weighting and calibration of segmental target frame rotation contribution). In upright position, sensitivity to IDPs were negligible (ΔIDP < 0.03 MPa) because IVRs and thus muscle activation patterns varied little. In flexion and extension, the influence on the calculated IDPs and IVRs were evident for the studied segmental rotation contributions of the target frames. As measured *in vivo* (Breen et al., 2021) non-linear segmental rotation contributions should be considered in the future given the relevance of our findings. Ligament strains, IDPs, or facet joint contact forces may represent alternative stability criteria besides the thorax target frame.

### 4.3 Validation

The validation of realistic simulation models requires experimental confirmation. *In vivo*, however, such data are difficult to acquire or may never be obtained. Given the large number of components and the inevitable combination of data from multiple sources, an absolute overall validation of our active hybrid model was not possible at this stage. Sole approach was the comparison of particular biomechanical model responses with experimental results under defined boundary conditions. Based on available publications, we therefore considered absolute IDP and IAP values and relative changes in muscle forces in 13 LCs. Moreover, to increase model validity, we extensively validated the passive LSS with experimental *in vitro* studies in all spatial directions in advance (Remus et al., 2021). This included ROMs, IVRs, IDPs, facet joint contact forces, instantaneous centers of rotation, functional spinal unit stiffnesses, and intervertebral disc bulges. This was done under the assumption of component validation (Lewandowski, 1982), based on the fact that models consisting of well-validated submodels are likely to be valid. Advantages compared to a direct validation of the entire active model included the higher number of useable *in vitro* studies, simplified loading conditions, and the influence analysis of LSS structures in case of a stepwise reduction of the anatomy (Heuer et al., 2007). Even though *in vivo* loading conditions cannot be correctly mimicked using a moment and follower load (Khoddam-Khorasani et al., 2018), muscle activation patterns represent boundary conditions too complex for fundamental, and detailed validation of the basic biomechanical responses of passive LSSs. The validation in this study was limited to symmetrical LCs in the sagittal plane. As other simulation models of the LSS (Liu et al., 2018; Malakoutian et al., 2018) have mostly been validated with the same *in vivo* studies as we have used, there are no significant differences in the biomechanical model responses. For more comprehensive model validation, additional movements such as axial rotations and lateral flexions as well as non-symmetric loads like holding a weight laterally and combined movements outside the anatomical planes need to be investigated.

#### 4.3.1 Muscle activation pattern

The most common approaches to distinguish for biomechanical muscle redundancy problems are optimization-driven and EMG-driven models (Mohammadi et al., 2015; Dreischarf et al., 2016). In EMG-driven models, muscle activities recorded via electrodes from *in vivo* measurements are used to solve the present redundancy problem (Knapik et al., 2012). If the solution of muscular redundancy is purely mathematical, it is an optimization-driven approach. The basic assumption is that at least one cost function is optimized by the central nervous system, while equilibrium constraints as well as upper and lower limits for muscle forces are fulfilled (Dreischarf et al., 2016). EMG-based optimizations combine both approaches to account for vital equilibrium constraints in addition to physiological EMG signals from human subjects to improve model predictions (Mohammadi et al., 2015). Solution methods for computing muscle forces in musculoskeletal models include forward and inverse optimization and optimal control (Erdemir et al., 2007; Stavness, 2010; Malakoutian et al., 2018). We used an optimization-driven approach to determine the muscle forces through which the spine is moved and stabilized. Considering equilibrium constraints via five target frames, the muscle activation patterns were calculated by the TC (Stavness, 2010; Stavness et al., 2010) integrated in ArtiSynth. Due to the redundancy of the muscle activities, the optimization problem was underdetermined (Stavness et al., 2010). The additional consideration of orientation constraints via target frames and muscle activity changes resulted in a multicriteria optimization. Despite the quadratic consideration of muscle cross-sections to select an efficient activation pattern, it is possible that only local optima for muscle activation patterns were found.

The muscle parameters of the two reference models (Christophy et al., 2012; Bayoglu et al., 2017) were based on *in vitro* studies and estimations. The parameters for pennation angle, passive stiffness, sarcomere length, and specific muscle tension were constant for most of the muscle groups. However, a recent simulation study (Malakoutian et al., 2022) showed the relevance of these paraspinal muscle parameters on the predicted spinal loads. To use reference material parameters, we scaled these linearly based on muscle cross-sections and body weight. Due to this, as well as the assumption of same parameters for the most muscle groups, a systematic error in our simulation results could not be excluded. We did not investigate possible correlations in more detail. Sensitivity studies with muscle group-specific parameters should therefore be pursued. Muscle activities determined were sensitive to varying weighting parameters. These were chosen iteratively in advance in such a way that all the LCs examined were solved in a stable manner and considered relative to one another, the weighting factors for the following terms were minimal with descending relevance: vertebral targets, muscle damping, thorax target, and muscle excitation. For a qualitative validation of the predicted muscle forces we used published EMG data, because *in vivo* measured muscle forces are generally not available (Erdemir et al., 2007). High agreement was shown in the relative changes of the muscle groups for all simulated LCs. Compared to another simulation study (Arshad et al., 2016), the sum of our local muscle forces (IL, LL, PM, MF, QL) was only slightly lower at about 272 N in the upright posture (N0_1_). With 30° flexion of the upper body, the estimated local muscle force tripled comparably. In flexion, moreover, the passive muscle force component became greater because the sarcomere lengths were thus extended.

#### 4.3.2 Intra-abdominal pressure

Compression of the abdominal cavity between the diaphragm and the pelvic floor is primarily regulated by activation of the enclosing abdominal musculature TA, EO, and IO (Goel and Weinstein, 1990). For validation, we computed all LCs without and with the predominantly transverse TA. Reason for this was the combination of the geometric and anatomical reference muscle data from two independent sources. It should be noted that the conversion dimensioning between the forces *F_IAP_* and *F_AP_* was performed only w/TA. The influence of TA turned out to be dependent on the LC considered and the biomechanical model response. It could be seen that IAP w/oTA was considerably underestimated compared to w/TA. Difficulty for validation was caused by limited *in vivo* data with a wide range of measured values. These span from 1.5 to 7.5 mmHg for upright standing without load held (Andersson et al., 1977; Schultz et al., 1982; Nachemson et al., 1986), which was probably measured most frequently. Measured values also differed by BMI (Cobb et al., 2005) and sex (Essendrop and Schibye, 2004). The percent increase in IDPs calculated in the model w/oTA was greater in upright posture than in flexion and extension. Comparable to another simulation study (Liu et al., 2019b), for w/TA and the resulting elevated IAP at greater flexion angles, E.S. activation as a global muscle group was reduced and IDP was reduced by up to 1.8%. In general, our results on the IDPs, which were in agreement with previous findings (McGill and Norman, 1987; Kumar, 1997; Arjmand and Shirazi-Adl, 2006b), suggest that the unloading function of the IAP is not to be overestimated. Much of the pressure was generated by the abdominal muscles, but they also had a compressive effect on the lower back. There is no way to contract the abdominal muscles without increasing the IAP (Cholewicki et al., 2002). In view of the effects on the other muscle groups as well as the current implementation, we considered TA to be a relevant muscle group in the simulation of sagittal-symmetric LCs. Furthermore, due to its role as a moment generator around the longitudinal axis (Cresswell et al., 1992), the relevance of TA in axial rotation and asymmetric LCs, is of particular importance. Consideration of the TA is also important for the examination of individuals with low back pain, for which this muscle group is often much more active (Knapik et al., 2022).

We attributed the underestimation of IAP in flexion and lifting activities (Figure 11) primarily to the fact that no explicit consideration of co-activation of the abdominal musculature was integrated as part of the solution of the muscle redundancy problem. Therefore, abdominal muscle activity also tended to be underestimated in other models with an optimization approach (Arjmand and Shirazi-Adl, 2006c; Liu et al., 2018). To overcome this, constant activations (Khoddam-Khorasani et al., 2020) or situation-dependent lower activity limits (El-Rich et al., 2004) for the abdominal muscles can be implemented in the future. Alternatively, the force *F*_*IAP*_, which results mathematically from the compressive forces of the abdominal muscles, can be considered as an additional variable target when solving the muscle redundancy problem. This can result in the abdominal muscles being more activated by the TC to reach a targeted IAP, depending on the upper body posture and the weighting in the cost function.

#### 4.3.3 Intradiscal pressure

*In vivo* IDP measurements (Schultz et al., 1982; Sato et al., 1999; Wilke et al., 2001; Takahashi et al., 2006) are useful and as mentioned before one of the few ways to gain quantitative insight into the biomechanics of the lumbosacral spine. The pressure directly reflects the load acting on the spinal level in question (Nachemson, 1981). In addition to the body’s dead weight and external masses, stabilizing muscular forces are also an integral part of the load. Therefore, the use of *in vivo* IDP measurements for the validation of simulation models was all the more relevant. In our model, the intervertebral discs were modeled as healthy and non-degenerated fiber-reinforced FE bodies with almost incompressible hyperelastic material behaviors. Assuming that the nucleus pulposus within the annulus fibrosus behaves hydrostatically throughout its volume (Nachemson, 1960), we calculated the IDP from the negative mean of the normal stresses of all FE nodes inside a nucleus pulposus (Remus et al., 2021). This assumption implies that *in vivo* only non-degenerated intervertebral discs can be reliably measured by means of a pressure transducer, because the proteoglycan-water gel must be present and mobile to a sufficient extent (McNally and Adams, 1992; Wilke et al., 2001). Degenerative alterations as well as the presence of collagen fibers in the gel that can cause anisotropic pressure distributions (McNally and Adams, 1992) are possible reasons for the partially considerably varying IDP in the literature. In general, IDP decreases when the intervertebral disc degenerates (Sato et al., 1999). The number of studies that measured IDP *in vivo* in different situations, however, was limited and primarily restricted to the levels L3/4 and L4/5. Because no suitable data were present for cranial lumbar spine levels and L1 and T12 were rigidly connected in the model, we considered only the three caudal spine levels in the validation. As in many biomechanical studies (Tafazzol et al., 2014), the thoracic region was represented as a single lumped rigid body in the current mode. Despite resulting variations in muscle forces, the influence on lumbosacral model responses was shown to be small in a simulation study (Ignasiak et al., 2016).

In comparison with the *in vivo* IDPs visualized in Figure 9, we found predominantly high agreement with the values calculated by our model. Only in upright position with 5 kg in each hand and without load were there relevant differences. In both cases, the model overestimated the IDP at L3/4 and L4/5. This did not apply to other LCs in the upright posture. As mentioned above, however, it was possible that the IDP measured by pressure transducers were reduced by degenerative changes or anisotropies. Also, not further traceable relieving postures of the examined subjects in upright standing could be plausible. However, especially the comparison with the measured values of Wilke et al. (2001), which were measured at L4/5 in a volunteer similar in body weight and height to the model, showed a very high agreement in all LCs. In general, the predicted load on the lumbar spine increased as the magnitude of segmental rotations increased, which was the case in both extension and flexion. Among others, also Andersson et al. (1977) and Nachemson (1965) observed an increase in pressure of comparable magnitude for flexions without, as well as with additional weights in the hands. The comparatively sharper increase in IDPs at 30° flexion we attributed to the fixed pelvis in the model. This is a limitation compared to other models (Liu et al., 2019a; Favier et al., 2021a; Honegger et al., 2021) that take into account a lumbopelvic ratio for larger movements and more activities. *In vivo* studies had shown (Tafazzol et al., 2014) that the upper body flexion is characterized by a simultaneous rotation of vertebrae and pelvis. The contribution of pelvis rotations increased with larger flexions. Small flexions were predominantly accomplished by vertebral rotations. A lumbopelvic rhythm should therefore be implemented in the future, especially for larger flexion angles.

## 5 Conclusion

This work is a new approach to computational spine biomechanics that combines optimization-driven trunk musculature, FE intervertebral discs, ligaments, and facet joints in one model. The resulting capability to use the interplay of passive and neurally coordinated active mechanisms to simulate spinal stability represents an advance on the state-of-the-art. Despite the discussed simplifications, the realized muscle-driven forward dynamic active hybrid model enables valid, robust, and efficient estimation of biomechanical responses of the lumbosacral spine under *in vivo* similar loads. Furthermore, the findings motivate a future application of the presented methods to develop patient-specific and pathological active hybrid spine models to study more complex load cases. This can be of therapeutic interest to identify conditions with great deflections but little stress on the intervertebral discs. More individualized therapies for muscle disorders through targeted strengthening or unloading are also conceivable. However, an important field of research will be the understanding and correlation to clinically relevant pain conditions.

## Data Availability

The original contributions presented in the study are included in the article/Supplementary Material, further inquiries can be directed to the corresponding author.

## Supplementary material

Supplementary Material S1 Tables with the locations of the COMs applied to the models’ components and polynomial curve fit coefficients *s*_*i*_ for the marker LAL41 DTW reference curves.

Supplementary Material S2 Virtual palpation dictionary for identifying anatomical characteristics of the human LSS, pelvis, abdomen, and thorax.

Supplementary Material S3 Illustrated description of palpated target anatomies including general remarks and descriptions for the workflow in NMSBuilder.

Supplementary Material S4 Registration atlas to facilitate the workflow by providing all LM names per bone in sorted order.

Supplementary Material S5 Muscle modeling parameters used in the model with the muscle attachments in the global coordinate system for the right side of the body.

Supplementary Material S6 Video showing LC F30_3_ for exemplary visualization of the forward dynamic simulation procedure with target frames and without musculature in right lateral view.
