# Supplementary material for: Muscle-driven forward dynamic active hybrid model of the lumbosacral spine: combined FEM and multibody simulation
Source: Front Bioeng Biotechnol. 2023 Sep 27;11:1223007. doi: 10.3389/fbioe.2023.1223007 (PMC10565495; doi:10.3389/fbioe.2023.1223007)
Supplement: Supplementary file 1 [file DataSheet2.PDF]

Virtual palpation dictionary for the registration process of lumbosacral spine bone landmarks (LMs)

by the author Robin Remus, Ruhr-University Bochum, 2022

LM number	LM name	van Sint Jan 2007	Schmid et al. 2017	Description
**Lumbar vertebrae (L1-L5)**				
Point 1	**LAV/S**			LAV superior - Superior side of lamina arcus vertebrae at the posterior tip of the vertebral foramen
Point 2	**SAP/R**			SAP right - most posterior point of right superior articular process
Point 3	**SP/I**			SP inferior - most inferior point of spinous process (in sagittal plane)
Point 4	**SP/P**	LV	SPL1-5	SP posterior - most posterior point of spinous process (in sagittal plane)
Point 5	**VB/A**			VB anterior - Most posterior point of the concave area of the anterior face of the vertebral body (in sagittal plane)
Point 6	**VB/PI**			VB posterior inferior - most posterior and inferior point of vertebral body (in sagittal plane)
Point 7	**VB/PS**			VB posterior superior - most posterior and superior point of vertebral body (in sagittal plane)
Point 8	**VB/RI**			VB right lateral inferior - most inferior and right lateral point of vertebral body
Point 9	**VB/RS**			VB right lateral superior - most superior and right lateral point of vertebral body
**Sacrum**				
Point 1	**CS/PS**			CS posterior superior - Posterior superior point of outer margin of bony canalis sacralis (in sagittal plane)
Point 2	**CS/S**			CS in sagittal plane - Intersection of a straight line from the end points of the cornua sacralia with the sagittal plane
Point 3	**LSC/1R**			LSC right - Lateral sacrum crest at right side at first sacral foramen
Point 4	**LSC/3R**			LSC right - Lateral sacrum crest at right side at third sacral foramen
Point 5	**MSC/2**	SS2		Tip of median sacral crest closest/between second sacral foramen
Point 6	**PAS/R**			PAS right - Most right and superior point of proc. articularis superior
**Pelvis**				
Point 1	**AC/R**	IAC		AC right - Center of right acetabulum
Point 2	**SIAS/R**	IAS	RTAS	SIAS right - Right spina iliac anterior superior
Point 3	**SIPS/R**	IPS	RTPS	SIPS right - Right spina iliaca posterior superior
Point 4	**CI/SR**	ICT	RTMS	CI superior right - Most superior point of the right crista iliaca measured from a reference line between SIAS and SIPS
Point 5	**SI/R**			SI right - Spina ischiadica at the right side
Point 6	**SN/R**			SN right - Tip of the right greater sciatic notch
Point 7	**TP/R**	IPP		TP right - Right tuberculum pubicum

Thorax

Point 1	**AC/7R**			AC 7 right - Angulus costae of the right 7th rib (fascies externa, inferior edge)
Point 2	**AC/9R**			AC 9 right - Angulus costae of the right 9th rib (fascies externa, inferior edge)
Point 3	**AC/11R**			AC 11 right - Angulus costae of the right 11th rib (fascies externa, inferior edge)
Point 4	**CC/7R**	RA7		CC 7 right - Anterior end of 7th right corpus costae (fascies externa)
Point 5	**CC/11R**			CC 11 right - Most inferior point of the 11th right corpus costae and/or cartilago costalis (fascies externa)
Point 6	**CS/I**	SXS		CS inferior - Most inferior point of the corpus sterni (in sagittal plane, anterior side)
Point 7	**JN**	SJN	CLAV	Jugular notch
Point 8	**SP/3P**	TV3	SPT1	SP T3 posterior - Most posterior point of 3rd thoracic spinous process (in sagittal plane)
Point 9	**SP/5P**	TV5	SPT2	SP T5 posterior - Most posterior point of 5th thoracic spinous process (in sagittal plane)
Point 10	**SP/7P**	TV7	SPT3	SP T7 posterior - Most posterior point of 7th thoracic spinous process (in sagittal plane)
Point 11	**SP/9P**	TV9	SPT4	SP T9 posterior - Most posterior point of 9th thoracic spinous process (in sagittal plane)
Point 12	**SP/11P**	TV11	SPT5	SP T11 posterior - Most posterior point of 11th thoracic spinous process (in sagittal plane)
Point 13	**TC/7R**			TC 7 right - Tuberculum costae of right 7th costa (rib)
Point 14	**TC/9R**			TC 9 right - Tuberculum costae of right 9th costa (rib)
Point 15	**TC/11R**			TC 11 right - Tuberculum costae of right 11th costa (rib)

Abdomen

Point 1	**CS/I**			CS inferior - Most inferior point of the corpus sterni (in sagittal plane, anterior side)
Point 2	**LA/L3**			LA L3 - Point at the linea alba which is a copy of SPP from L3 shiftet in horizontal plane to the center of the abdominal ellipsoid
Point 3	**LA/L4**			LA L4 - Point at the linea alba which is a copy of SPP from L4 shiftet in horizontal plane to the center of the abdominal ellipsoid
Point 4	**SIAS/R**			SIAS right - Right spina iliac anterior superior
Point 5	**TP/R**			TP right - Right tuberculum pubicum

Humerus

Point 1	**CTMI/R**			CTMI right - Right crista tuberculi minoris directly below at the collum chirurgicum
Point 2	**HH/R**			HH right - Center of humeral head
Point 3	**TMA/R**	HGT		TMA right - Right tuberculum major (greater tubercle)
Point 4	**TMI/R**	HLT		TMI right - Right tuberculum minus (less tubercle)
