# Supplementary material for: Muscle-driven forward dynamic active hybrid model of the lumbosacral spine: combined FEM and multibody simulation
Source: Front Bioeng Biotechnol. 2023 Sep 27;11:1223007. doi: 10.3389/fbioe.2023.1223007 (PMC10565495; doi:10.3389/fbioe.2023.1223007)

Documentation and visual explanation of the virtual palpation process to register anatomic landmarks (LMs) to the lumbosacral bones

v1.2 (19.07.2022)

Robin Remus, Ruhr-University Bochum – remus@lpe.rub.de

General remarks

This documentation presents a brief overview of the relevant aspects of a virtual palpation and the specifics for an LSS model. For more details, please refer to the documentation of the publication by Modenese et al. 2018 (https://doi.org/10.1016/j.jbiomech.2018.03.039)

- The palpation process is performed with NMSBuilder 2.1 (http://www.nmsbuilder.org/)
- For symmetry reasons, the landmarks are currently applied only to the right sides of the following bones: Thorax, Humerus, L1, L2, L3, L4, L5, Sacrum, and Pelvis
- To register insertion points of the abdominal muscles, especially connected to the linea alba, an abdominal plate must be palpated. However, in this case all anatomic landmarks are dependent on the surrounding bones (Thorax, L3, L4, and Pelvis)
- For the following visualisations a modified anatomy of the Male VHP is used which is described in more detail for the lumbosacral spine in Remus et al. 2021 (https://doi.org/10.1371/journal.pone.0250456)

Tips and Tricks for the workflow

- Select the appropriate bone and press ***Ctrl + A*** to access the *Operations* tab and <u>add</u> or <u>edit</u> landmarks
  - When adding new landmarks to the model load the dictionary **Virtual_registration_atlas.dic** via the GUI and select the current bone via the drop down menu
- To modify a landmarks (LMs) position e.g. shift from a vertebral body to the abdominal plate, select the LM by clicking on it and press ***Ctrl + T***
  - To move a LM within a plane go to the tab *View settings,* select the matching view (e.g. lateral view) from one of the six views, and switch back to the *Operation* tab.
  - Change the reference system via the drop down menu to *VME Reference System* and pull the correct arrow to move the LM in plane.
  - **<u>Warning</u>**: Do not exit the transformation dialog by any other input than the steps listed before. End the successful transformation only with *Ok* or use *Cancel*. Otherwise, NMSBuilder may crash and the model must be reloaded from the last save point.

Overview of the skeletal model

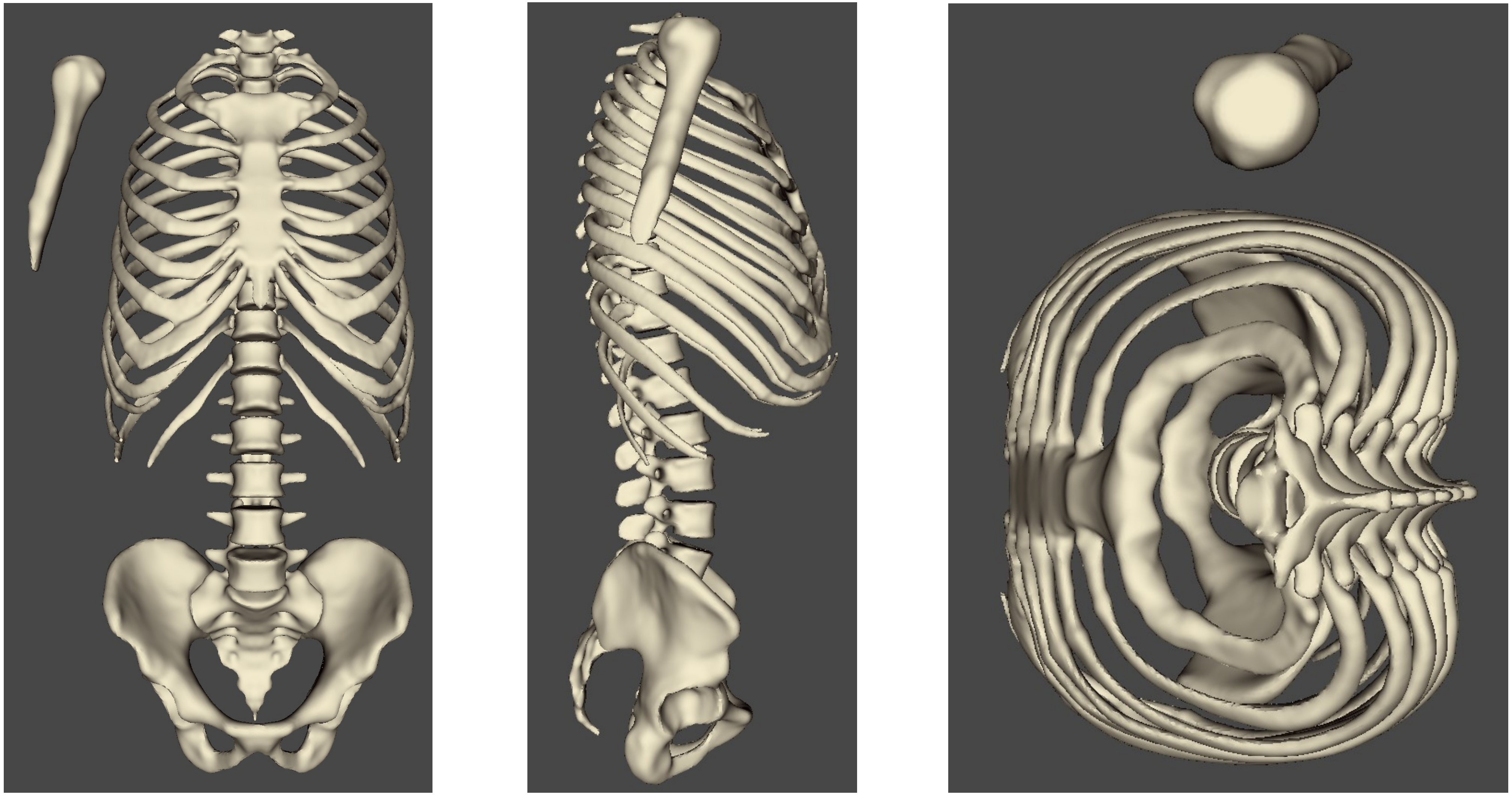

Vertebrae L1 – L5 (L2 is depicted)

LM	
#1	LAVS
#2	SAPR
#3	SPI
#4	SPP
#5	VBA
#6	VBPI
#7	VBPS
#8	VBRI
#9	VBRS

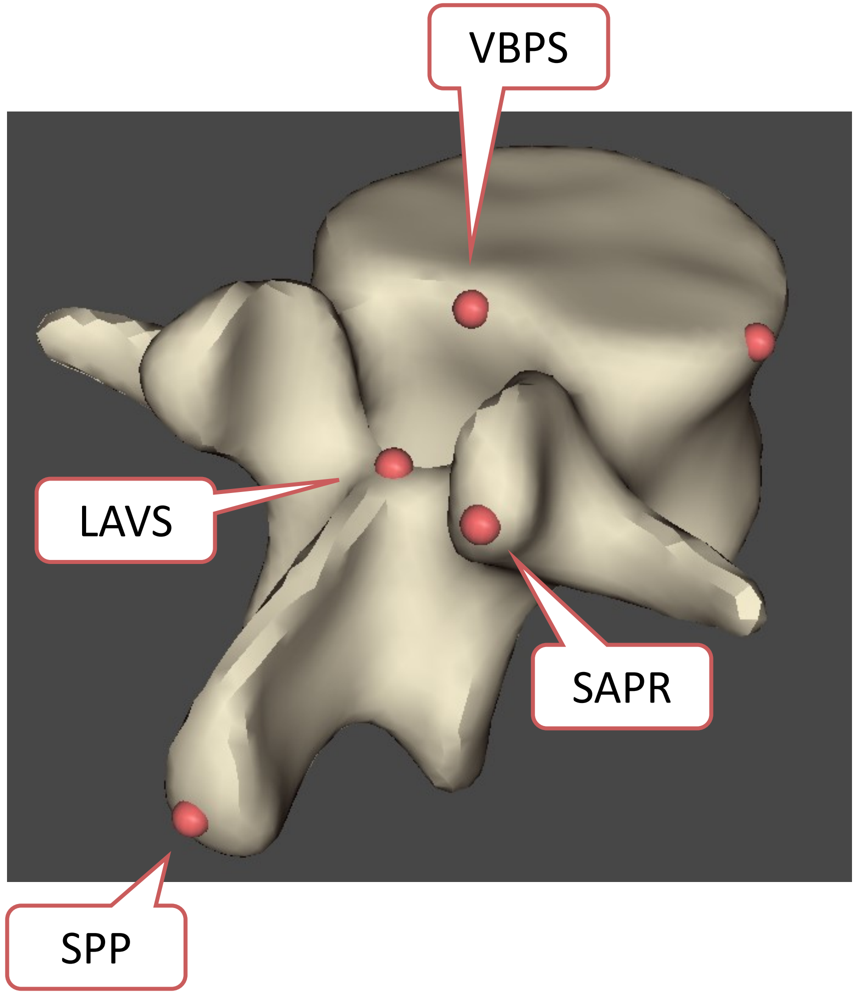

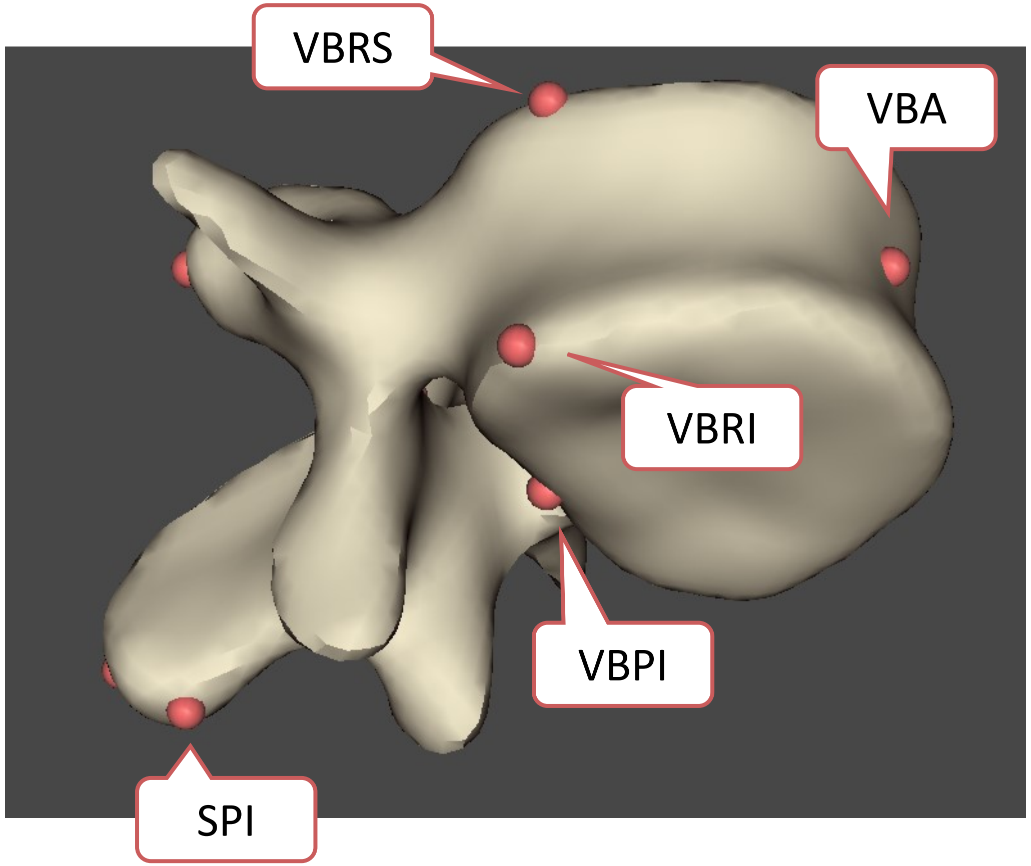

Sacrum

LM	
#1	CSPS
#2	CSS
#3	LSC1R
#4	LSC3R
#5	MSC2
#6	PASR

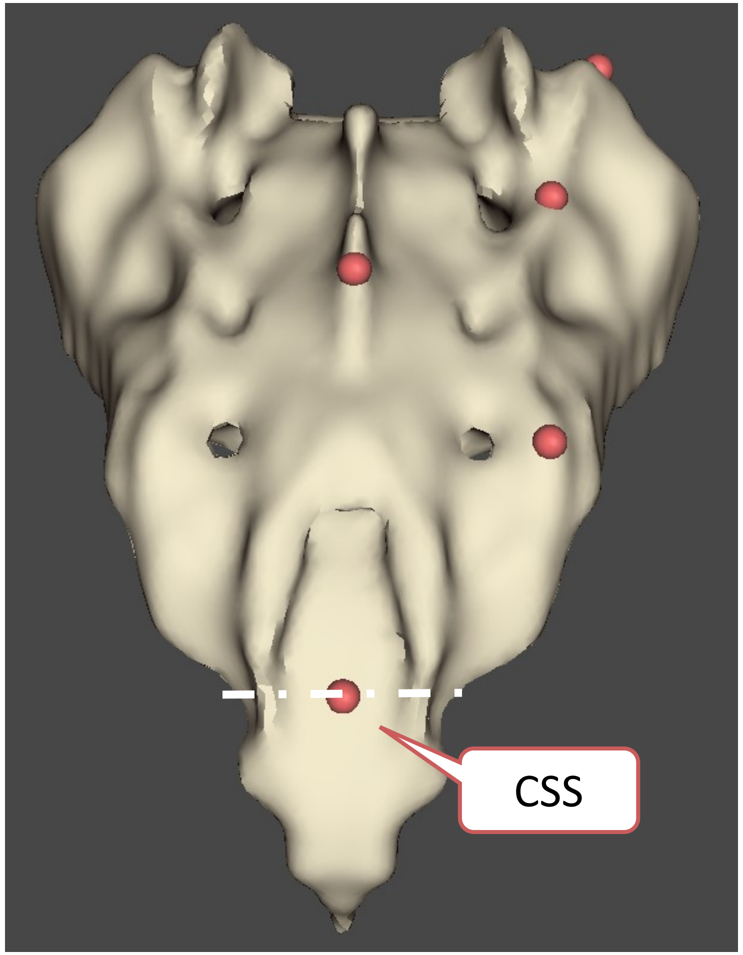

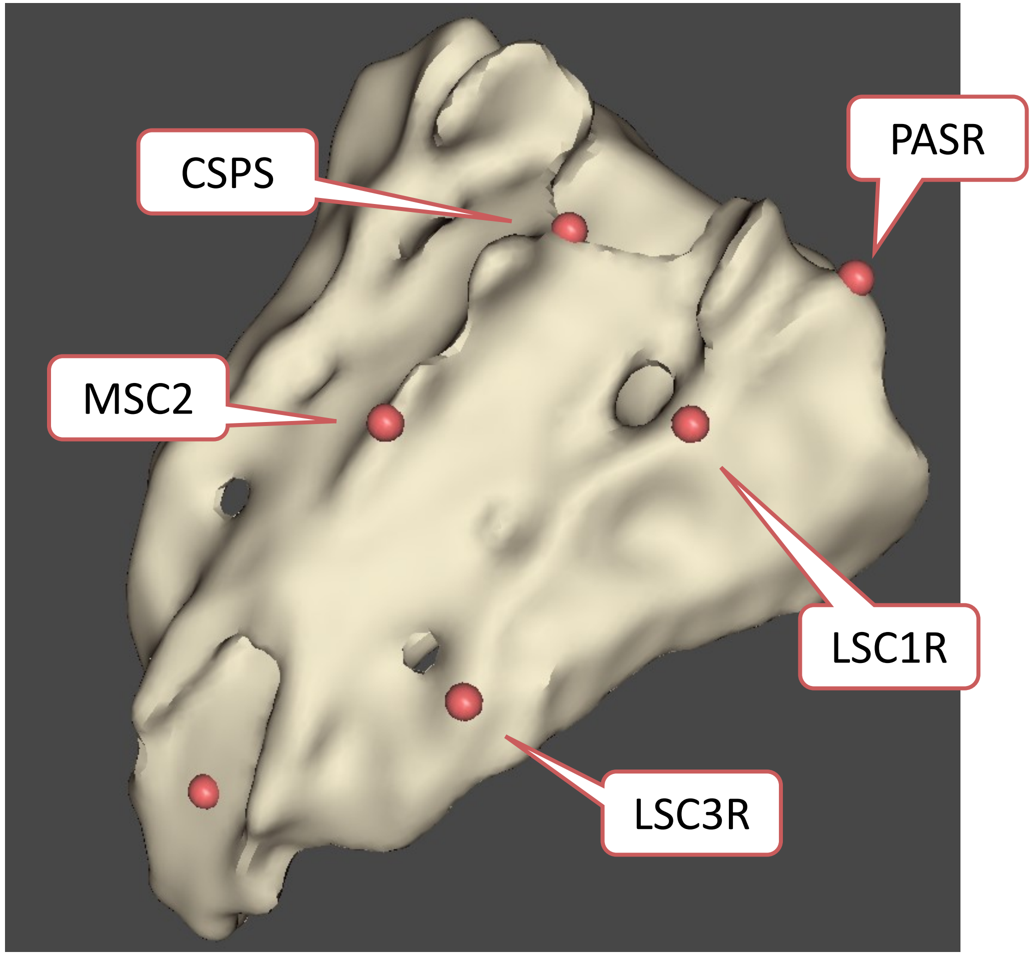

Pelvis

LM	
#1	ACR
#2	CISR
#3	SIASR
#4	SIPSR
#5	SIR
#6	SNR
#7	TPR

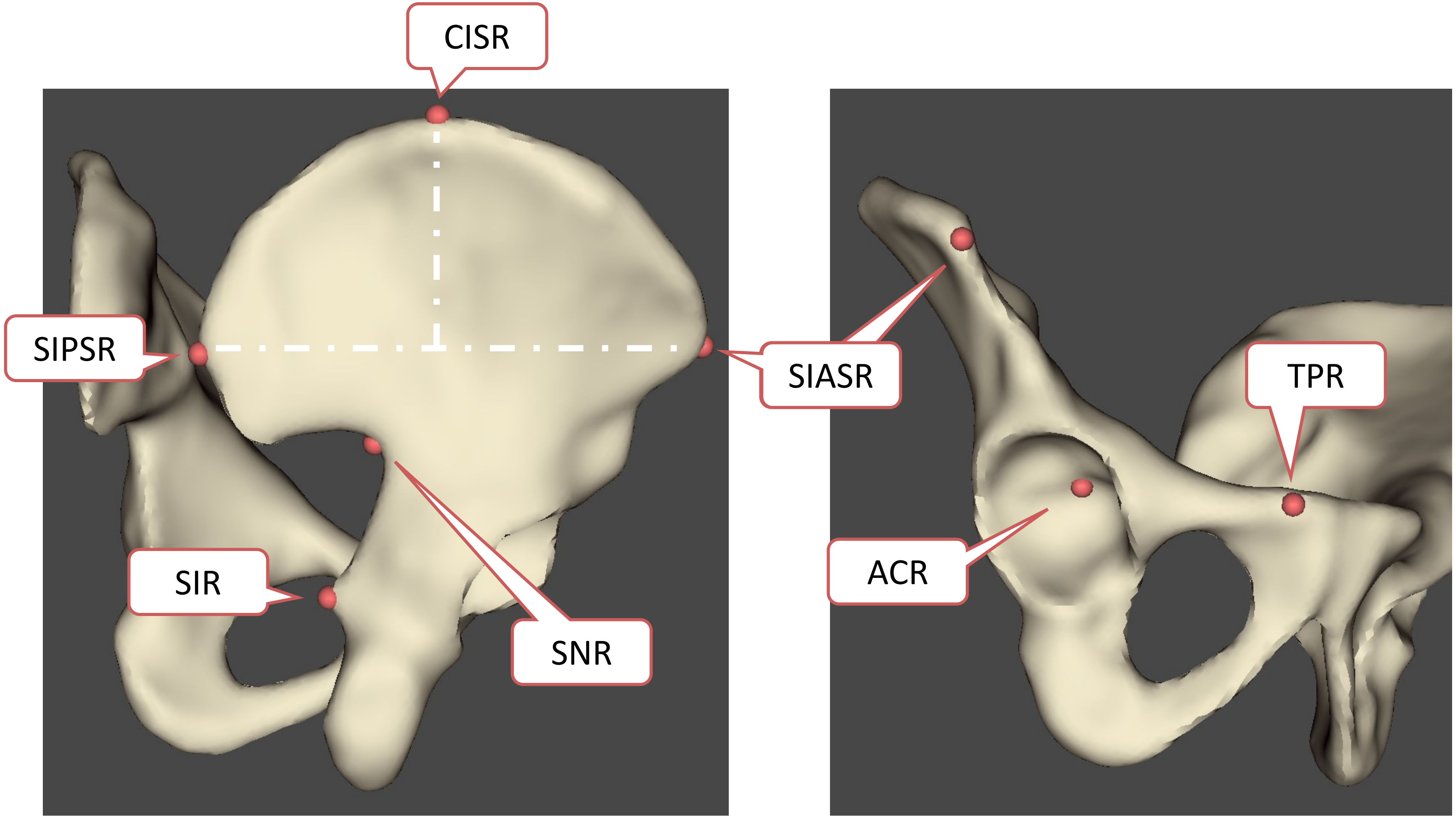

Thorax

#1	AC7R
#2	AC9R
#3	AC11R
#4	CC7R
#5	CC11R
#6	CSI
#7	JN
#8	SP3P
#9	SP5P
#10	SP7P
#11	SP9P
#12	SP11P
#13	TC7R
#14	TC9R
#15	TC11R

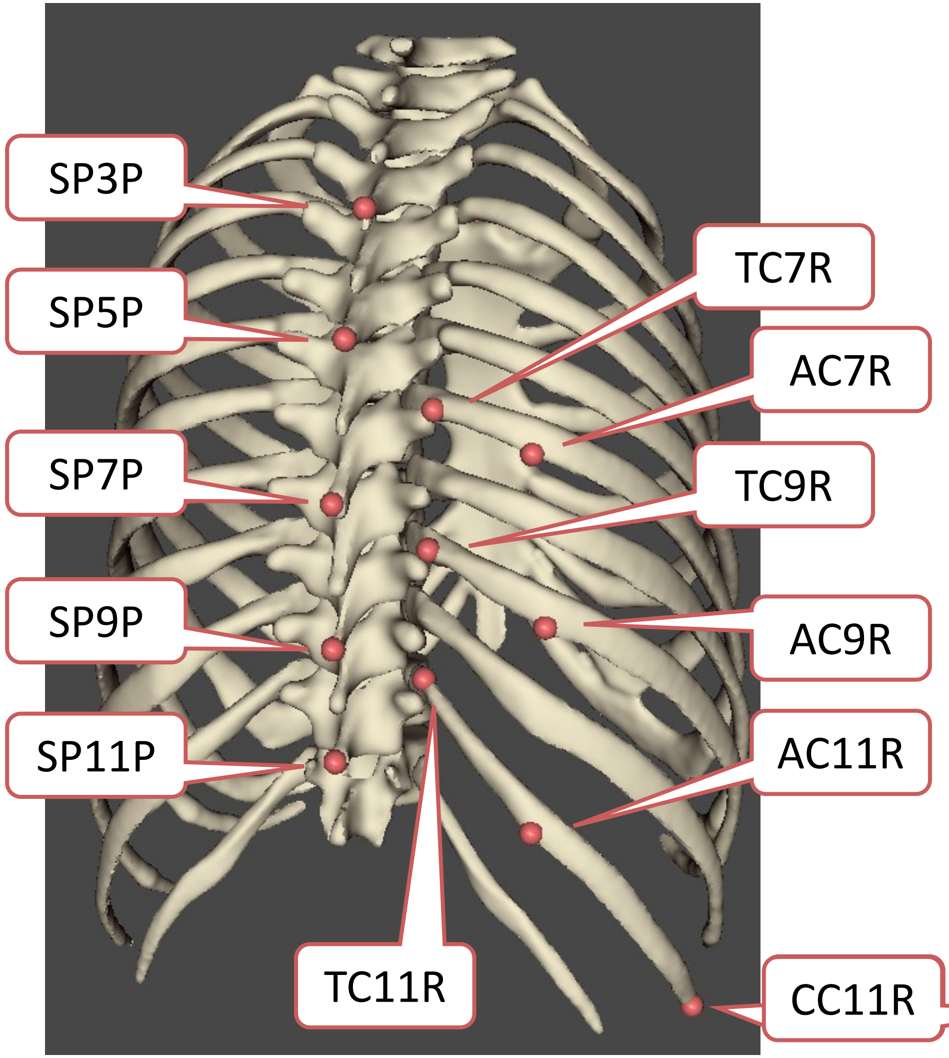

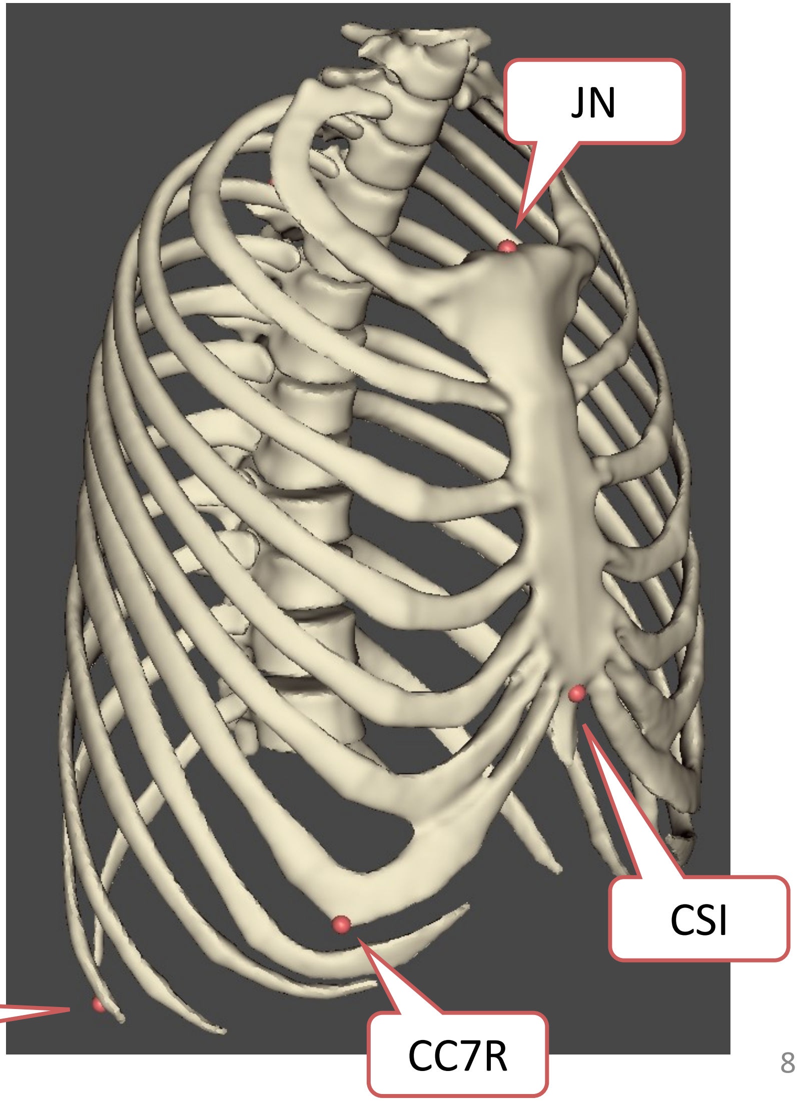

(Right) Humerus

LM	
#1	CTMIR
#2	HHR
#3	TMAR
#4	TMIR

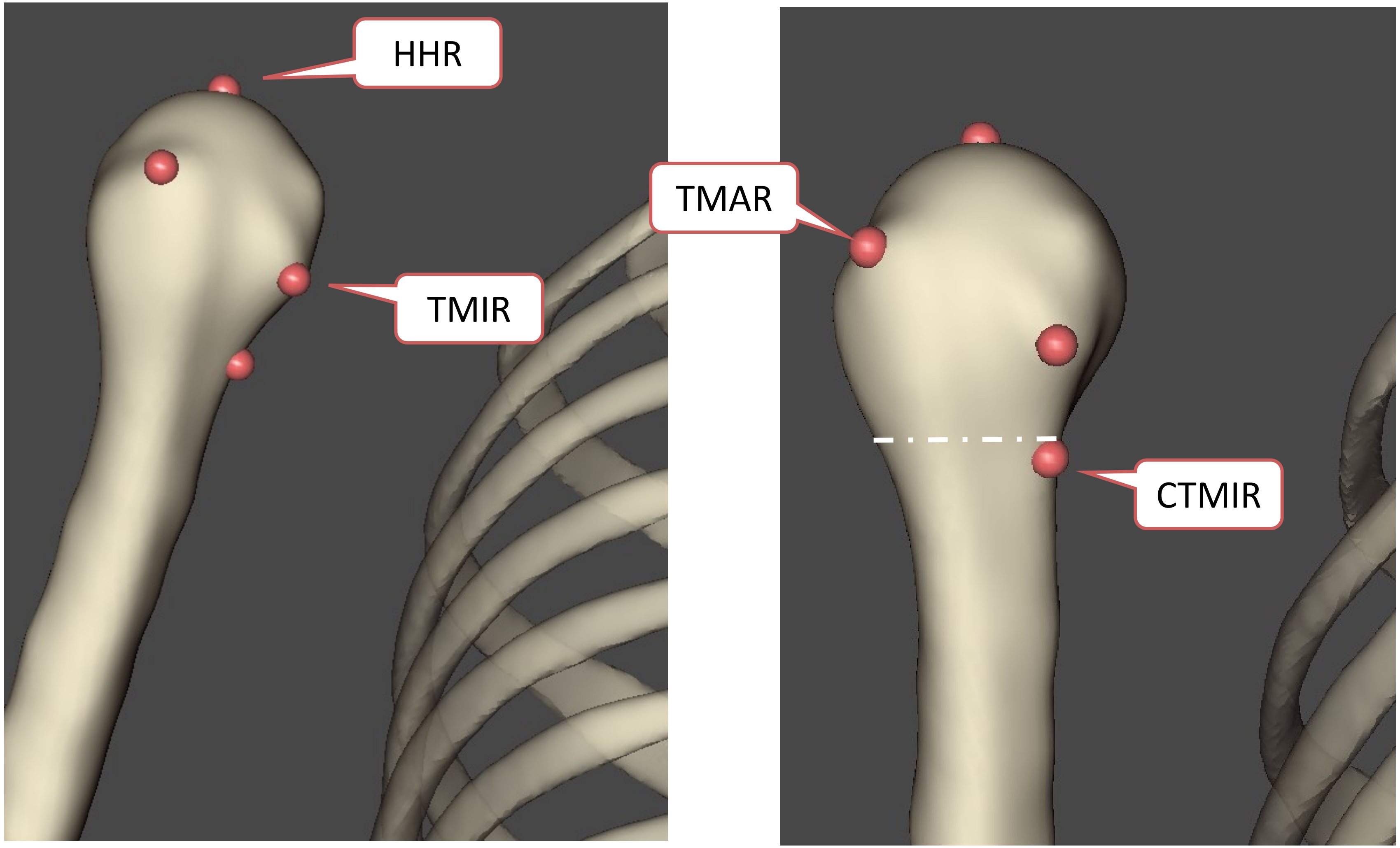

Abdominal Ellipsoid

LM	
#1	CSI
#2	LAL3
#3	LAL4
#4	SIASR
#5	TPR

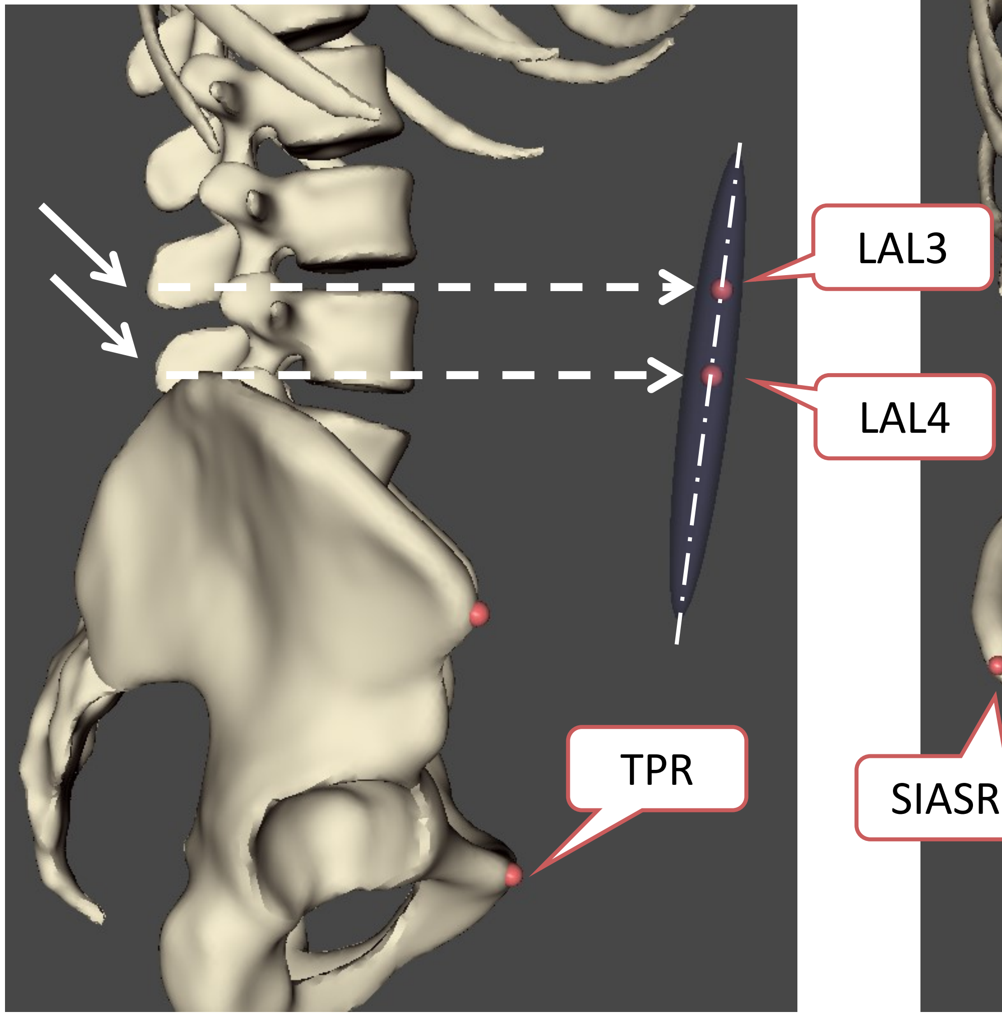

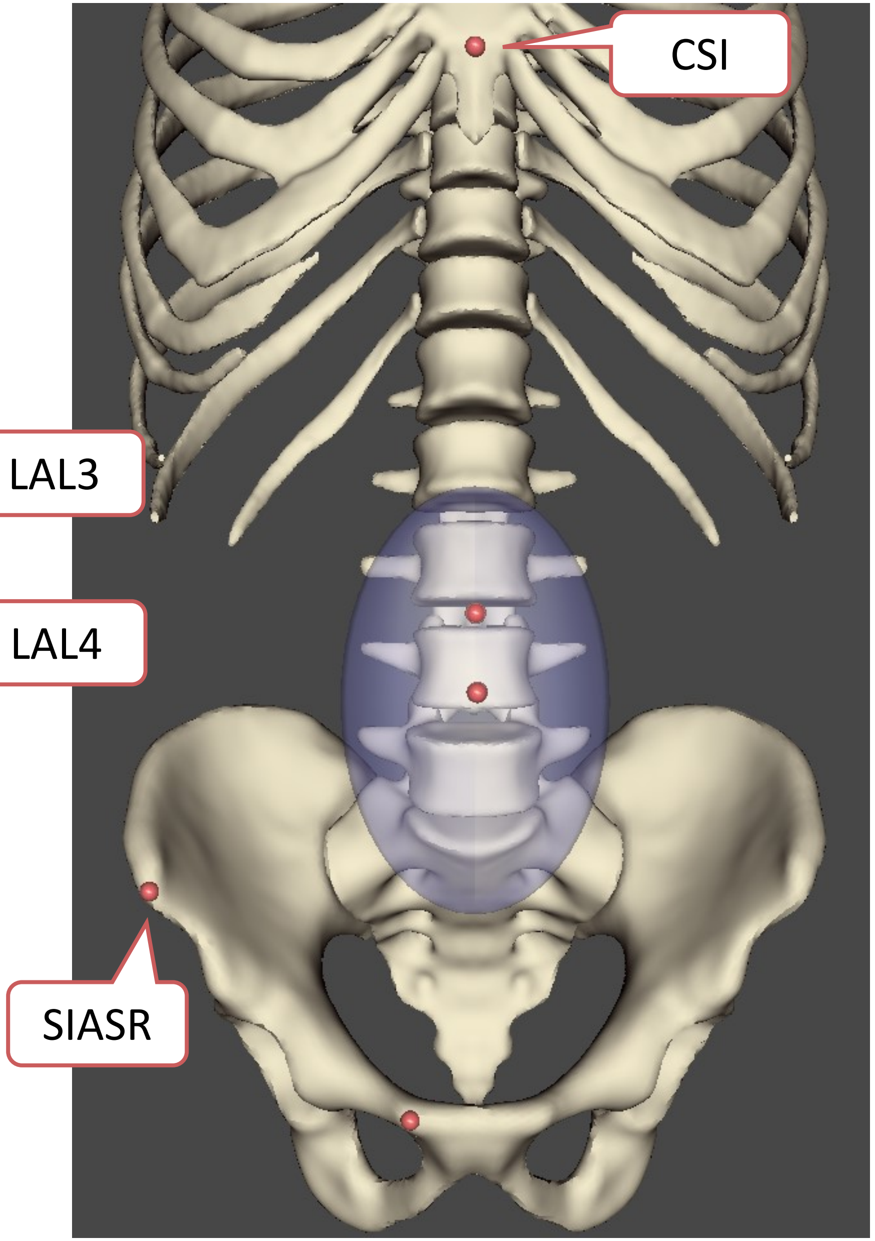

Supplement: Supplementary file 3 [file DataSheet3.PDF]
