# Supplementary material for: Muscle-driven forward dynamic active hybrid model of the lumbosacral spine: combined FEM and multibody simulation
Source: Front Bioeng Biotechnol. 2023 Sep 27;11:1223007. doi: 10.3389/fbioe.2023.1223007 (PMC10565495; doi:10.3389/fbioe.2023.1223007)
Supplement: Supplementary file 4 [file DataSheet1.pdf]

Supplementary Material I

Table A Locations of the COMs applied to the models' components in present global coordinate system. The body mass of the entire male VHP is $b = 90$ kg. The masses given were taken from literature (Vette et al., 2011).

Label	Model component	Location *x*, *y*, *z* (m)	Mass (kg)
**COM_{UB}**	Thorax	-0.0054, 0.0, 0.3729	36.44
**COM_{L1}**	L1	0.0314, 0.0, 0.1839	2.621
**COM_{L2}**	L2	0.0327, 0.0, 0.1447	2.541
**COM_{L3}**	L3	0.0276, 0.0, 0.1051	2.624
**COM_{L4}**	L4	0.0218, 0.0, 0.0635	2.520
**COM_{L5}**	L5	0.0081, 0.0, 0.0254	2.588

Table B Polynomial curve fit coefficients s_i for the marker LAL41 DTW reference curves, describing the principal movements from upright standing to the ROM limit of the thorax.

**Movement**	**Projected coordinates**	**ROM limit (deg)**	**Polynomial parameters**				
			**Degree**	s_1	s_2	s_3	s_4
Flexion	*x*	30	1	19.233	-2.137		
	z		3	19.468	-80.341	0.171	0.061
Extension	*x*	20	3	2.267	-19.964	1.936	0.476
	z		3	-0.261	13.314	12.772	-0.380
Axial Rotation	*x*	10	1	-1.536	0.128		
	y		2	-4.289	-15.168	0.830	
Lateral Bending	*y*	15	2	8.035	0.855	-0.172	
	z		2	0.217	-2.307	0.388	

REFERENCES

Vette, A. H., Yoshida, T., Thrasher, T. A., Masani, K., and Popovic, M. R. (2011). A complete, non-lumped, and verifiable set of upper body segment parameters for three-dimensional dynamic modeling. *Med Eng Phys* 33, 70–79. doi: 10.1016/j.medengphy.2010.09.008
